# Inhibitory control tests in non‐human animals: validity, reliability, and perspectives

**DOI:** 10.1111/brv.70055

**Published:** 2025-07-28

**Authors:** Louise Loyant, Luke Collins, Marine Joly

**Affiliations:** ^1^ Department of Psychology, Centre for Comparative and Evolutionary Psychology University of Portsmouth King Henry Building, King Henry 1 Street Portsmouth PO1 2DY UK

**Keywords:** self‐control, animal cognition, executive control, response inhibition, cognitive control, behavioural inhibition, battery of tasks, motor regulation

## Abstract

Inhibitory control, the ability to control impulsive or pre‐learned behaviour in order to reach a more rewarding goal, is essential in many aspects of normal life. In non‐human animals, better inhibitory control performances have been associated with a larger brain, better problem‐solving skills, and fitness benefits. This crucial cognitive ability has been studied in a wide range of fields (psychology, neurosciences, animal cognition) and has been tested in several animal classes from insects to mammals. Unfortunately, unlike in human test psychology, the common paradigms designed to measure inhibitory control in non‐human animals often suffer from a lack of validity and reliability and have yielded mixed results. Therefore, the nature of inhibitory control, either defined as a common ability or a suite of distinct processes, is still debated. Besides, the evolutionary processes shaping the variation in inhibitory control, often tested using a single task, are still poorly understood and the relative influences of ecological, anatomical or social factors as evolutionary drivers of this ability remain unclear. Finally, it is only recently that researchers have focused efforts on the factors necessary for the evolution of inhibitory control, that is individual variation in inhibitory control performance, heritability of this trait and fitness benefits. Hence, our main objective herein is to conduct a review of the existing literature to discuss conceptual and methodological challenges faced by researchers wanting to study inhibitory control in animals. We then suggest tools to tackle these challenges and propose a framework to build a valid and reliable measure of inhibitory processes. Next, we describe the requirements to study the selective pressures involved in this cognitive process in order to have a better understanding of its evolutionary underpinnings. We finally consider the future of interspecies comparative studies of inhibitory control.

## I. INTRODUCTION

Inhibitory control is defined as the ability to control impulsive or pre‐learned behaviours in order to reach a more rewarding goal (Diamond, 2013; Macleod, 2007; Nigg, 2017). A strong internal predisposition or an external distractor that is inappropriate to the individual's goal must be overridden in order to do what is more appropriate or needed (Diamond, 2013; Dillon & Pizzagalli, 2007; Nigg, 2017). For instance, in the wild, stalking predators such as wolves must inhibit premature movements and maintain restraint while approaching prey, delaying the pounce until the optimal moment for a successful capture (MacNulty, Mech & Smith, 2007). In social groups, low‐ranked individuals must override mating or feeding activities in front of more dominant groupmates (Amici *et al*., 2018; Byrne & Bates, 2007). Hence, inhibitory control allows animals to respond to the environment in a flexible manner and adjust behaviours that may be counterproductive or potentially harmful.

Inhibitory control is part of executive functions – higher cognitive processes that support goal‐directed action (Banich, 2009; Friedman & Miyake, 2017). The main inhibitory processes presented in the literature are the inhibition of attention to a distraction (e.g. control of an internal or external interference), the inhibition of an action (e.g. a reflexive motor action is withheld or withdrawn) and inhibition of a pre‐learned behaviour [e.g. ability to adjust behaviours flexibly in the context of dynamically changing goals (Diamond, 2013; Dillon & Pizzagalli, 2007; Nigg, 2017)]. Here, we emphasise the discrimination between inhibitory control and self‐control, which are terms that are often confused in the literature (see Beran, 2015). In a self‐control task, there must be two options available that are differentially preferred by the subject and there must be a cost to obtaining the more preferred outcome (Beran, 2015). As it requires decision‐making, self‐control is considered a more advanced cognitive ability within the umbrella term of inhibitory control (Beran, 2015, 2018). Delayed gratification tasks (waiting for a better reward) are examples of self‐control tasks (e.g. Miller *et al*., 2019).

In humans, inhibitory processes have been associated with greater intelligence (e.g. quantified by the IQ test; Duckworth & Seligman, 2005) and positive life outcomes (Moffitt *et al*., 2011; Tangney, Baumeister & Boone, 2004). In animals, inhibitory control performances have been associated with brain size (Horschler *et al*., 2019; MacLean *et al*., 2014; Stevens, 2014), problem‐solving skills (Ashton *et al*., 2018; Hauser *et al*., 2002; Müller *et al*., 2016; Vlamings, Hare & Call, 2010) or fitness benefits (Ashton *et al*., 2018; Boogert *et al*., 2011; Shaw, 2017). Such inhibitory processes, crucial in any individual's day‐to‐day life, could thus be one of the components essential to respond to problems in a flexible manner, which might be particularly important for rapidly changing social environments (Hauser *et al*., 2002; Müller *et al*., 2016; Vlamings *et al*., 2010). As these findings are correlational, it could also be possible that there is a common underlying factor that affects both inhibitory control and problem‐solving abilities.

Even though inhibitory control has been studied in a wide range of fields, including psychology, neurosciences, ethology, and animal cognition, and has been tested in several animal classes such as insects, fishes, reptiles, birds and mammals, the nature of inhibitory control is still debated. Historically considered as a general ability, researchers now propose inhibitory control as a family of distinct components (Friedman & Miyake, 2017; MacLeod, 2007; Nigg, 2017). However, methodological issues have been reported recently in tasks commonly used to measure inhibitory control, leading to unreliable results and inconsistencies between inhibitory control tasks (van Horik *et al*., 2018a; Beran, 2018; Völter *et al*., 2018).

Finally, despite the pivotal role of inhibitory control, the evolutionary processes shaping variation in inhibitory performance remain poorly understood. Some authors argue than inhibitory control could have evolved through ecological (MacLean *et al*., 2014; Amici, Aureli & Call, 2008), anatomical (MacLean *et al*., 2014; Stevens, 2014) or social pressures (Johnson‐Ulrich & Holekamp, 2020; Ashton *et al*., 2018). For instance, it has been hypothesised that as brains get absolutely larger, the increasing total number of neurons creates the potential for new functional areas involved in inhibitory functions (MacLean *et al*., 2014). Moreover, it has been hypothesised that inhibitory abilities are essential for living in more complex social groups, as inappropriate impulsive behaviours would be disadvantageous in cooperative relationships (Amici *et al*., 2008; Dunbar & Shultz, 2007). Inhibitory control would also be adaptive for foraging challenges: individuals with the highest cognitive flexibility may be able to explore new dietary resources or more efficient methods of food acquisition (MacLean *et al*., 2014). However, some of the proposed evolutionary factors have been challenged by recent studies (Jelbert, Taylor & Gray, 2016; Kabadayi *et al*., 2017b) as animals with smaller brains showed a similar performance to larger‐brained species in inhibitory control tasks.

Progress in understanding inhibitory control has been hampered by practical and conceptual challenges, including difficulty in defining this ability and challenges to validation of appropriate and reliable methods to quantify it and to demonstrate the necessary conditions for its evolution.

Hence, our main objective herein is to review the existing literature to discuss methodological and conceptual challenges faced by researchers studying inhibitory control in non‐human animals (hereafter referred to as animals). We conducted a systematic literature search following the PRISMA (Preferred Reporting Items for Systematic Reviews and Meta‐Analyses; Page *et al*., 2021) guidelines to identify studies investigating inhibitory control in non‐human animals. Our aim was to review methodological and conceptual challenges related to the assessment of inhibitory control, focusing on studies published between January 2000 and June 2023. The search was carried out using three databases: *PubMed*, *ISI Web of Science*, and *Google Scholar*. To identify relevant studies, we developed a search strategy combining key words related to inhibitory control and non‐human animal research. To refine the search further and focus on specific experimental paradigms commonly used to assess inhibitory control, we incorporated an additional set of task‐specific terms. The final search string, therefore, used Boolean combinations structured as follows: (“inhibitory control” OR “response inhibition” OR “motor self‐regulation” OR “behavioural inhibition” OR “cognitive control” OR “executive function” OR “attentional bias” OR “self‐control”) AND (“non‐human animals” OR “animal” OR “nonhuman primate” OR “monkey” OR “bird” OR “canid” OR “species”) AND (“detour task” OR “cylinder task” OR “barrier task” OR “reaching task” OR “Go/No‐go task” OR “reversal learning task” OR “A‐not‐B task” OR “Stroop task” OR “emotional Stroop task”). Search syntax was adjusted as necessary to meet the requirements of each database. Studies were included if they met the following criteria: they were published in English between 2000 and June 2023; they reported empirical data from at least one experimental task specifically designed to assess inhibitory control; they included at least one non‐human animal species; and they provided sufficient methodological information to allow evaluation of the task's design, validity, and reliability. We excluded articles that were review papers, meta‐analyses, or theoretical discussions without original data, as well as studies focusing exclusively on human participants or transgenic models. We also excluded studies in which inhibitory control was only tangentially addressed (e.g. as a minor component of a broader personality or temperament assessment), where the methodology did not permit specific conclusions about inhibitory processes (e.g. treatment or manipulation without a control) and where only non‐adult individuals were tested. The screening process was conducted in two stages. First, all records retrieved from the database searches were screened based on their titles and abstracts to exclude irrelevant studies. In the second stage, full‐text versions of potentially eligible articles were retrieved and independently assessed for inclusion by at least two authors. Any discrepancies were discussed among the authors until a consensus was reached. For inclusion in the quantitative analysis, studies were additionally required to report exact methodology with similar tasks or slightly modified ones, and performance metrics, such as accuracy or error rates, in order to allow direct comparison across studies. The articles identified by each stage of study selection are listed in Table S1 (see online Supporting Information), which details the number of records identified, screened, assessed for eligibility, and ultimately included in the review. A total of 413 records were retrieved through database searches, including 70 from PubMed, 143 from ISI Web of Science, and the first 200 results from Google Scholar. We screened the titles and considered 333 papers. After removing duplicates, 263 records remained. The full texts of these articles were assessed for eligibility. After careful evaluation, a total of 86 studies met all inclusion criteria and were included in the review. In addition to the systematic search, we included a set of 57 studies previously identified through manual screening of key journals in the area and citation tracking. These studies met the same eligibility criteria. In total, 143 papers were included in the review (Table S2).

Based on our evaluation of the reported methodologies, we recommend a framework for devising valid and reliable measures of inhibitory processes. We then examine the requirements to reach a better understanding of the evolutionary underpinnings of inhibitory control. Lastly, we consider collaborative projects as a crucial step for the future of interspecies comparison of inhibitory control skills.

## II. INHIBITORY CONTROL IN ANIMALS

### (1) Common tasks in animals

**Fig. 1:**
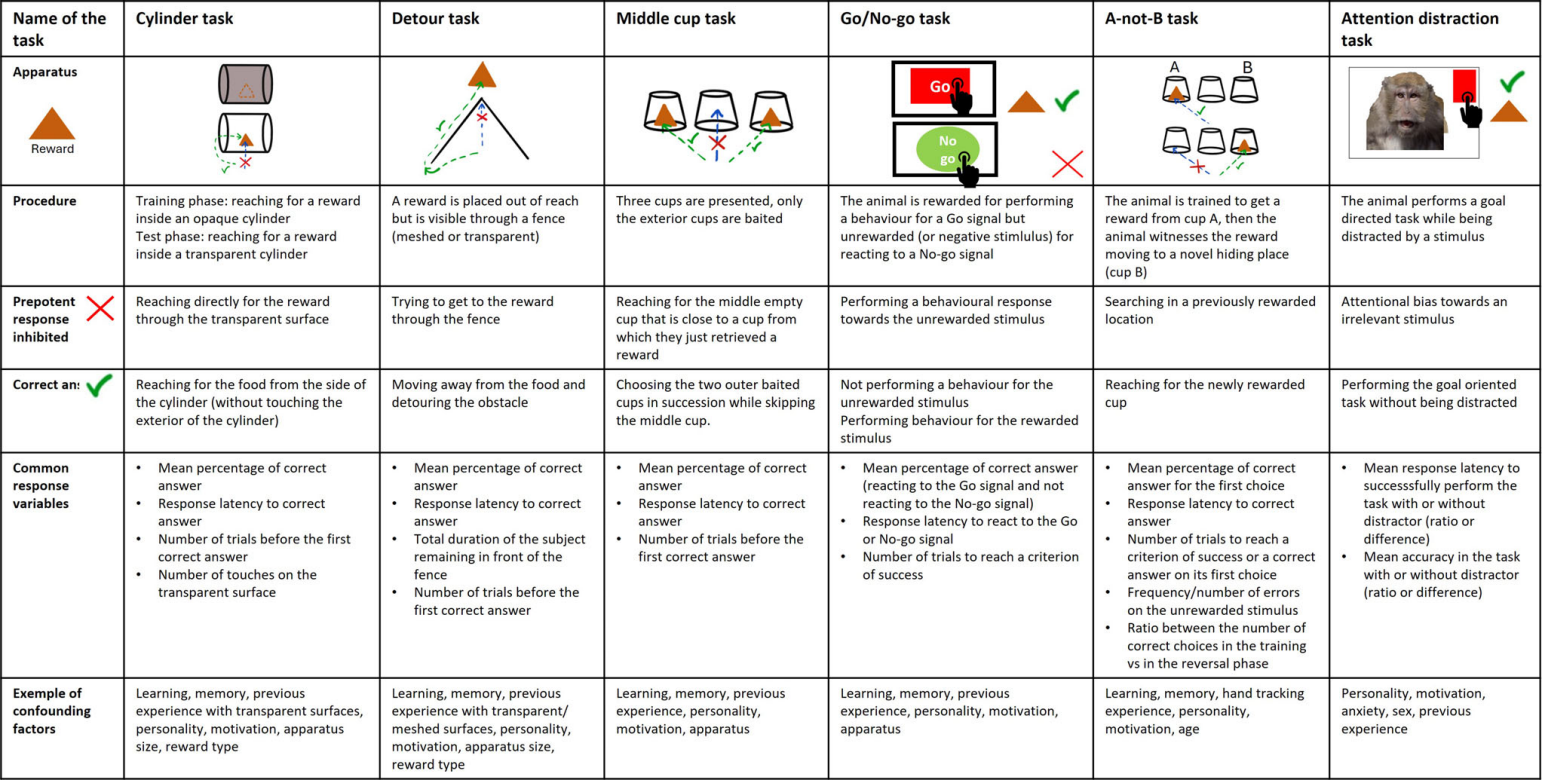
Common tasks of inhibitory control. A schematic representation of the apparatus is provided in the top row. For each task, the prepotent response inhibited, the correct response, the common response variables and examples of confounding factors are presented (see Table S4 for a detailed list of confounding factors and the direction of their effects). The A‐not‐B task is a simplified version of the Reversal learning task. We include tasks used in more than five papers in our review. The social inhibition task is not shown as the methods vary among papers.

**Fig. 2:**
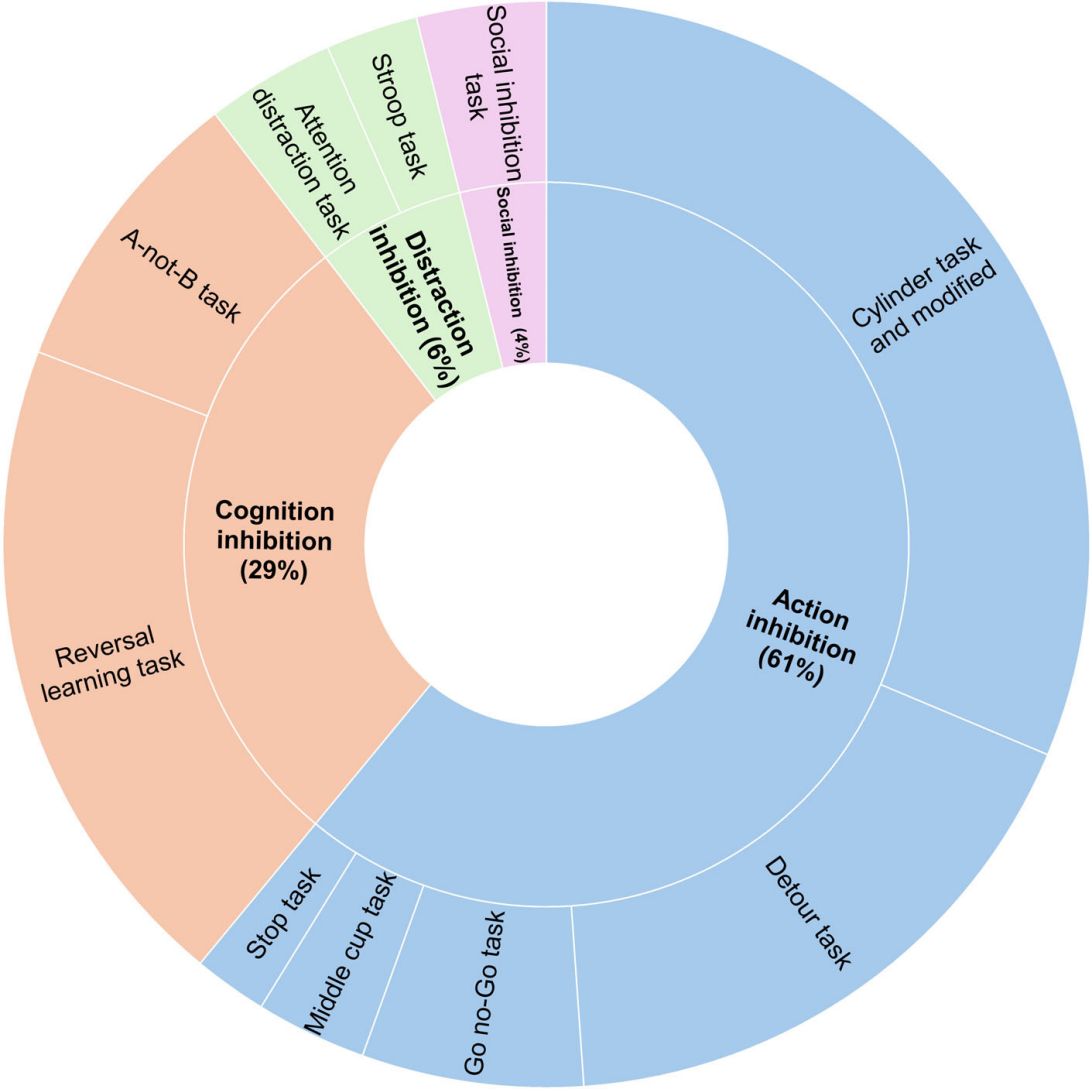
Proportion of each type of inhibitory control component (action inhibition in blue, distraction inhibition in green, cognition inhibition in orange and social inhibition in pink) measured in our sample of reviewed papers as a percentage of all tasks. A paper can contain different tasks. For clarity, each task is categorised according to its main task demands (inner ring) but note that a task can require more than one inhibition category.

Animal studies have mainly focused on tasks of inhibition of action (used in 61% out of 182 tasks in our sample of papers, see Figs 1 and 2).

In these tasks an impulsive motor response (prepotent response) needs to be inhibited in order to reach a goal (see Fig. 1). Often adapted from simplified tasks for children (for review see Petersen *et al*., 2016), these tasks are mainly used because of their simplicity and ease of implementation (Kabadayi, Bobrowicz & Osvath, 2018). The most common inhibitory control task of all domains combined is the cylinder task and its modifications (used in 31.3% of papers). This task was adapted from the detour reaching task primarily designed to study object permanence in infants (Piaget, 1954). In this task, infants have to inhibit their tendency to reach directly for a visible reward through a transparent barrier. Infants of 6–11 months have great difficulty inhibiting reaching directly for the reward (Diamond, 1990). For this cylinder task, animals are usually habituated to reach for food from the sides of an opaque cylinder [in Ashton *et al*. (2018) animals were not pre‐trained]. Subsequently the opaque cylinder is replaced by a transparent cylinder and the individuals must inhibit reaching directly for the food through the transparent barrier (for review see Kabadayi *et al*., 2018; see Fig. 1). A variant of the cylinder task is the swing door task, in which a reward is placed behind one of two doors but attempts to reach it by pushing the door make the reward fall backwards and out of reach. This task similarly tests whether subjects can refrain from reaching for the reward directly and instead must open the non‐baited door to access the reward from behind (Amici *et al*., 2008; Vlamings *et al*., 2010). The reward can also be placed inside a transparent box (Brucks *et al*., 2017), behind a Plexiglas sheet (Amici *et al*., 2008) and was adapted for fish (guppies, *Poecilia reticulata*) by placing the reward inside a glass tube (Lucon‐Xiccato, Montalbano & Bertolucci, 2020b). The cylinder task is a variant of the more general detour task. In the detour task (used in 17.6% of papers), the reward is usually behind a fence (V‐ or U‐shaped) or a transparent wall and the animal needs to move around this obstacle (Marshall‐Pescini, Virányi & Range, 2015; Vernouillet *et al*., 2018; see Fig. 1). Another less‐common measure of the inhibition of an action is the middle cup task (used in 3.3% of papers), in which three cups are presented to the subject with only the cups from the extremities baited. The subject needs to inhibit directly reaching for the closest cup, the middle one (see Fig. 1). The Go/No‐go task (used in 6.6% of our sample of papers) and its variant the stop task (used in 2.2% of our sample of papers) are used less frequently in animal research as extensive training is necessary. In the Go/No‐go task (Gordon & Caramazza, 1982) the subjects need to respond to frequent Go stimuli while withholding a prepotent response to infrequently presented No‐go stimuli (Diamond, 2013; see Fig. 1). In the stop task, often confused with the Go/No‐go task, the subject similarly needs to respond to “stop” signals. However, in this task subjects are required to stop mid‐way through an already initiated trial (Diamond, 2013). This task is commonly used to measure the ability to inhibit a dominant motor response. Meier *et al*. (2017) developed a variation of the stop task with no extensive training required in which pheasants were required to adjust their movement in space in pursuit of a reward with a changing location. Finally, the five‐choice serial‐reaction time task (5CSRTT), mainly used in rodents, measures how the subject learns to respond to a stimulus displayed at one of five random locations. Touching or pointing to a location that did not show the target is regarded as a deficit in inhibitory control (Fletcher *et al*., 2007; Robbins & Crockett, 2010).

For the inhibition of cognition (or the inhibition of a pre‐learned rule), researchers commonly use the reversal learning task (used in 19.8% of the tasks), a simplified version of the Wisconsin card sorting task used to assess cognitive reasoning, attentional set shifting or more generally executive function (Grant & Berg, 1948). This reversal learning task challenges both the ability to inhibit a previously successful behaviour and the ability to produce a second behaviour in response to the same stimulus (Griffin, Guillette & Healy, 2015). In the reversal learning task the subjects first learn a stimulus–reward contingency; once a pre‐specified criterion is reached, this first association is reversed. Subjects must thus inhibit a previously correct response and shift their responses to a new stimulus–reward contingency (Bond, Kamil & Balda, 2007; Tapp *et al*., 2003). The A‐not‐B task (a simplified version of the reversal learning task, used in 8.8% of papers) originated from Piaget's (1954) study on the development of object permanence in human infants. A reward is hidden in the same location multiple times and the subject learns to find it here each time (acquisition phase). Afterwards, the reward is hidden in another location while the subject is watching (reversal phase). The animal then needs to adjust its behaviour by inhibiting its previous knowledge and reaching for the reward in the second location (see Fig. 1).

Another task occasionally used in animals to study inhibition of distraction is a variant of the Stroop task (used in 2.7% of papers). The Stroop task (Stroop, 1935) is one of the oldest tasks used in human psychology to demonstrate cognitive interference when incongruent stimuli are presented. A “Stroop effect” refers to the increased amount of time it takes to name the colour of a word when the ink colour and the word are incongruent. In animals, the Stroop interference effect has mainly been studied in non‐human primates. For instance, in rhesus macaques (*Macaca mulatta*), researchers used a numerical version of the Stroop task (Washburn, 1994. In another study, chimpanzees (*Pan troglodytes*) were tested with an incongruent association between a symbol and colour (Beran, Washburn & Rumbaugh, 2007). In a different chimpanzee study, subjects had to choose a stimulus based on its colour but the frame around the stimulus was from the alternative colour (Allritz, Call & Borkenau, 2016). The attention distraction task is a more recent variant of the Stroop task which does not involve a stimulus conflict but rather captures attention and variation in response time due to the emotional valence of the stimulus (Ben‐Haim *et al*., 2016). These tasks measure the inhibition of an emotional external distractor which interferes with the goal of the task (see Fig. 1). The attention distraction task was mainly designed for non‐human primates. Researchers used a negative stimulus, such as a picture of a vet (Allritz *et al*., 2016), an animal handler with gloves and a net (Boggiani, Addessi & Schino, 2018), snakes (Shibasaki & Kawai, 2009), or a threatening conspecific face (Bethell *et al*., 2012, 2016; King *et al*., 2012; Lacreuse *et al*., 2013).

The capacity to inhibit behaviours in a social context (which we term social inhibition here, as the test is conducted in the presence of a social partner) has been relatively neglected in the literature (used in only 4% of papers). It can be assessed in an artificial context (for example, when engaging in a task, individuals must assess the social context and inhibit inappropriate behaviours depending on the social status of other conspecifics present) or in a more natural context such as eating. For example, in non‐human primate species, subordinate individuals wait for the dominant individual to leave before executing a behaviour, suppressing the prepotent response of eating when dominants are nearby (Amici, Call & Aureli, 2009). Social inhibition can also be triggered by the presence of a human. For instance, lemurs inhibited approaching a reward in the presence of a competitive experimenter (Reddy *et al*., 2015), and in another study, dogs (*Canis familiaris*) had to inhibit their impulses to reach for a forbidden food while the owner was watching or not (Horschler *et al*., 2019).

### (2) Range of study subjects

**Fig. 3:**
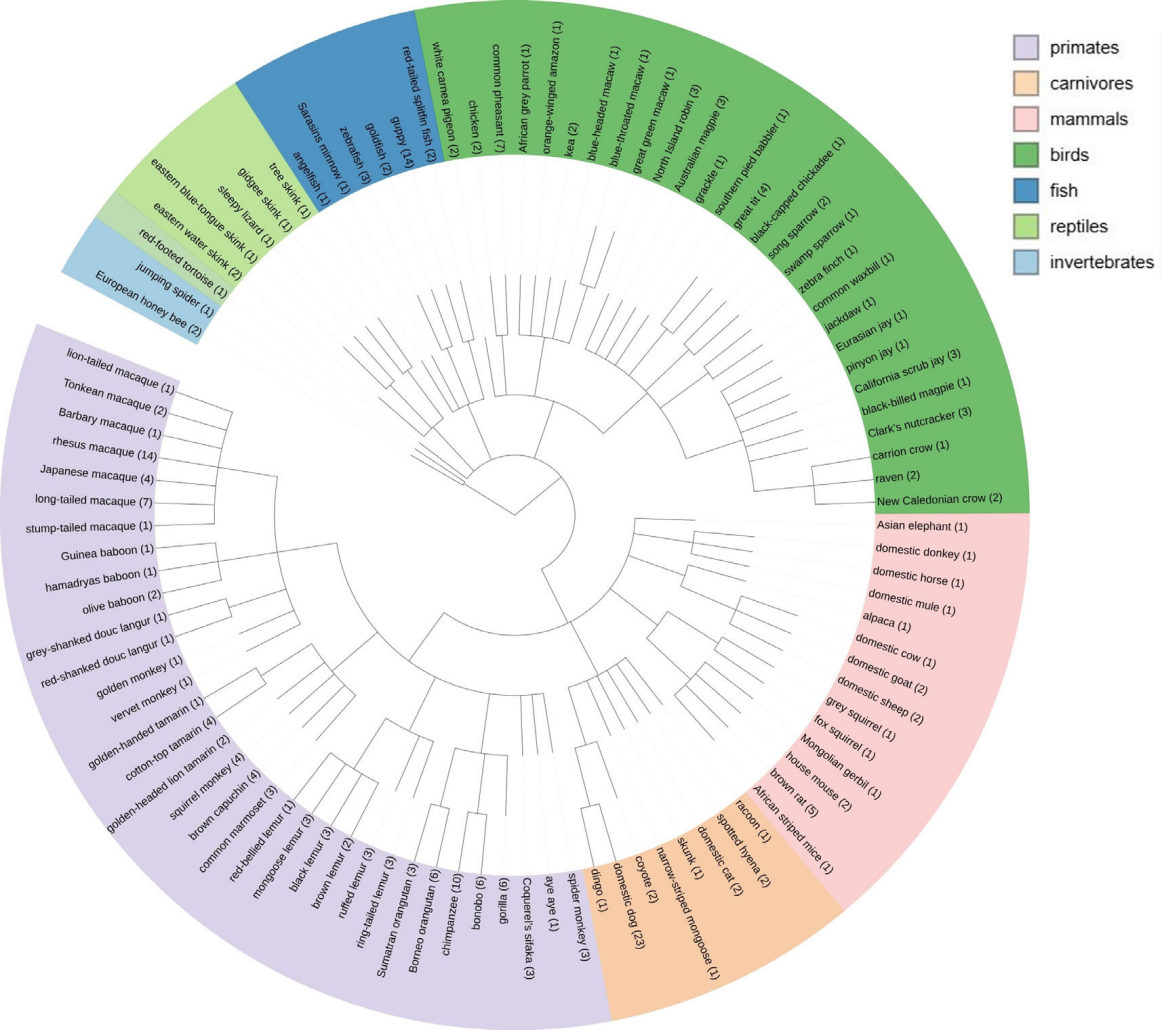
Phylogenetic tree of animal species tested in inhibitory control tasks. Numbers in parentheses indicate the number of articles using at least one task of inhibitory control. The same article can use different species. Tree created with PhyloT v2 (Letunic & Bork, 2024).

Inhibitory control has been studied in a wide range of non‐human species including invertebrates, fish, reptiles, birds and mammals (Fig. 3). In our sample of papers (Table S2), the primate order was the best represented (with 34 different species; Fig. 3), and rhesus macaques were the most common species tested (in 14 papers) perhaps due to their relative accessibility to researchers as a model used extensively in cognitive, neuroscientific and biomedical research (Phillips *et al*., 2014). Chimpanzees are also well represented (in 10 papers). The same bias for these two species was found in a meta‐analysis of primate cognition studies (ManyPrimates *et al*., 2019), which identified this sampling bias as problematic for deriving evolutionary inferences. For instance, rhesus macaques might have psychological characteristics that differ from under‐represented related macaque species, and that can affect their social cognitive skills (Joly *et al*., 2017). We simply do not have enough information on the other species of this taxon to assess whether rhesus performances are representative of macaque social cognitive skills. Dogs are also common subjects in studies of inhibitory control (23 studies; Table S2), perhaps due to recent enthusiasm from dog owners to participate in scientific experiments (Horschler *et al*., 2019). Dogs have been one of the most studied species during the last 20 years in research on comparative cognition (e.g. Bensky, Gosling & Sinn, 2013; Kaminski, 2008). Among birds, the common pheasant (*Phasianus colchicus*) is the most studied (used in seven papers, mainly from a single research group). For fishes, the guppy was used in 14 papers. Improved recent investigation of underrepresented taxa such as birds and fish could be due to advances in techniques for rearing, testing and housing large numbers of individuals.

## III. CHALLENGES IN MEASURING INHIBITORY CONTROL

### (1) Validity of inhibitory control tasks

#### (a) A neuroscientific approach

Surprisingly, despite their extensive use, few studies have assessed systematically the validity of measurements of inhibitory control, that is to demonstrate that the task is actually measuring inhibitory control performance. In neuroscience, it is common to infer mechanisms from neural underpinnings. Hence, one way to assess the validity of an inhibitory control task is to compare inhibitory performance of healthy subjects with performance of subjects with lesions of the frontal cortex, a region that is key to inhibitory control (e.g. Cook, Spivak & Berns, 2016). For example, one study demonstrated the validity of the stop task and the Go/No‐go task by matching performance of humans with specific brain lesions to that of healthy subjects (Thomas, Rao & Devi, 2016). In animal research, targeted brain lesions or electrophysiology have been used to study the neural mechanisms underlying inhibitory control. For instance, lesions of the prefrontal cortex induced impairment in the performance of common marmosets (*Callithrix jacchus*) on a detour reaching task (Wallis *et al*., 2001). Similarly in Japanese macaques (*Macaca fuscata*), electrical stimulation of these regions induced changes in performance in the Go/No‐go task (Sakagami *et al*., 2001). However, studies involving lesions or stimulation of a specific neural region and its consequences on inhibitory control should be interpreted with caution as each task will involve multiple abilities, and each ability is unlikely to be controlled by a single brain region. Due to ethical considerations, less‐intrusive paradigms, including functional magnetic resonance imaging (fMRI), positron emission tomography (PET) scans and electroencephalogram (EEG) technologies, are now increasingly adopted in animal studies (Cook *et al*., 2016; Sakagami *et al*., 2001). For example, Cook *et al*. (2016) used fMRI on conscious dogs performing a Go/No‐go task to localise the frontal brain regions underpinning response inhibition. In non‐mammalian animals, the association of particular brain regions with inhibitory control performances has been poorly studied. In fish, a larger telencephalon has been associated with better performance in a detour task in guppies (Triki & Bshary, 2021). More research is needed to understand better the neuronal underpinnings of inhibitory control in animals.

#### (b) The nature of inhibitory control and contextual validity

Another way to assess the validity of a task is to evaluate its contextual validity (or convergent validity), that is to compare the performance of subjects in several tasks thought to measure the same ability. If the subjects maintain their individual ranking across several tasks of inhibitory control, then researchers could hypothesise that the tasks are valid and effectively measure the targeted inhibitory ability. A major complication arises from the fact that the nature of inhibitory control is still much debated in both the neuropsychological and cognitive literature (Duckworth & Kern, 2011; Friedman & Miyake, 2004; Gärtner & Strobel, 2019; MacLeod, 2007; Miyake *et al*., 2000; Nigg, 2017). It remains unclear whether different tasks of inhibitory control are measuring the same underlying general inhibitory function, that is provide evidence for a unitary nature of inhibitory control, or if they are measuring independent inhibitory components, that is indicate a multifaceted structure.

In support of the unitary hypothesis of inhibitory control, a large‐scale meta‐analysis (including data from over 33,000 adult human participants), found a moderate but significant convergent validity of inhibition‐related measures such as the Go/no‐go task, the Stroop task and reversal learning paradigms (Duckworth & Kern, 2011). Similarly in animal research, a large comparative study found a strong positive correlation between the cylinder and the A‐not‐B task across 36 animal species (MacLean *et al*., 2014). MacLean *et al*. (2017) also found that the cylinder and two detour tasks loaded positively onto the same factor in a large‐scale battery of cognitive tasks in 552 dogs. Similarly, also in a study on dogs, performances in the A‐not‐B task were correlated positively with performances in the Go/No‐go task (Cook *et al*., 2016).

By contrast, several authors have proposed that inhibition‐related processes represent a family of functions rather than a single unitary construct (Friedman & Miyake, 2004, 2017; Nigg, 2000, 2017). In humans, Friedman & Miyake (2004), using common inhibitory control tasks, found that two inhibitory factors: “prepotent response inhibition” and “resistance to distractor interference” were closely related, but both were unrelated to “resistance to proactive interference”, a form of cognitive set inhibition. In the animal cognition literature, Völter *et al*. (2018), in a meta‐analysis using data on individuals from MacLean *et al*. (2014) did not find any correlation between the inhibitory control tasks. In the same meta‐analysis (Völter *et al*., 2018), analysis of the results from another battery of non‐human primate tasks (Amici *et al*., 2008) produced mixed results with only two significant correlations among four inhibitory control tasks (between the A‐not‐B and a variant of the cylinder task), in two out of three species. In another non‐human primate study, the performances of rhesus macaques in both a Modified Stroop Task and a Go/No‐go task were consistent, but not in the reversal learning task (Loyant *et al*., 2022). Similarly, several studies on dogs did not find significant correlation of performances between different inhibitory control tasks such as the detour task, the cylinder task, and the A‐not‐B task (Bray, MacLean & Hare, 2014; Brucks *et al*., 2017; Fagnani *et al*., 2016; Vernouillet *et al*., 2018). From these results, the authors concluded that inhibitory control is context specific and of a diverse structure (Bray *et al*., 2014; Brucks *et al*., 2017; Fagnani *et al*., 2016; Vernouillet *et al*., 2018).

This problem seems to be deep rooted: even performances in tasks thought to measure the same sub‐components are not coherent. For example, even among motor inhibition tasks the results were not correlated. In a study using the cylinder task and the detour task (putatively both measuring the inhibition of an action) in pheasants, performances on these tasks were not correlated (van Horik *et al*., 2018b). A similar lack of correlation was demonstrated for the cylinder task and the detour paradigm in grey wolves (*Canis lupus*) and dogs (Marshall‐Pescini *et al*., 2015). One straightforward explanation of this lack of consistency/correlation between tasks could be a lack of validity and reliability of these tasks (Dillon & Pizzagalli, 2007; Friedman & Miyake, 2004; Gärtner & Strobel, 2019; MacLeod, 2007; Völter *et al*., 2018). Indeed, researchers often assume that these tasks measure inhibitory control without providing any justification of their reliability, task choice or validity (Friedman & Miyake, 2004). More research aiming to validate batteries of inhibitory control tasks is needed to disentangle this lack of consistency in contextual validity.

### (2) Reliability of inhibitory control tasks

An explanation for the contradictory results and the null or weak correlations found might be a lack of test–retest reliability (also known as temporal repeatability). Unfortunately, common inhibitory control tasks tend to suffer from poor repeatability, that is multiple exposures to the same task often show inconsistent individual performance over time and context (Cauchoix *et al*., 2018; Friedman & Miyake, 2004; Völter *et al*., 2018). Unreliable tasks can attenuate correlations between measurements, even if the tasks measure the same ability (Völter *et al*., 2018). In this context, assertions made by researchers about the diverse structure of inhibitory control might be premature: assessing the reliability of a task is essential before considering its validity (Biro & Stamps, 2015; Friedman & Miyake, 2017; Griffin *et al*., 2015; Völter *et al*., 2018). This necessary step seems particularly relevant in the context of the “replication crisis” in psychology (Lindsay, 2015).

Some authors have made great efforts towards assessing the reliability of common inhibitory control tasks. A human research study investigated the temporal stability of measures of inhibitory control (such as the Go/No‐go task, the stop task and the Stroop task), and found good temporal reliability for most of the tasks (Wöstmann *et al*., 2013). Similarly, two studies demonstrated temporal reliability of the Go/No‐go task and the stop task (Weafer, Baggott & de Wit, 2013; Thomas *et al*., 2016). In the animal literature, a few papers have specifically assessed the temporal reliability of cognitive tasks. One meta‐analysis investigated the repeatability of cognitive performances in animals and found a moderate reliability of tasks related to learning, memory and physical cognition (Cauchoix *et al*., 2018). A recent study on four species of great apes (*Gorilla gorilla*, *Pongo abelii*, *Pan paniscus*, *P. troglodytes*) demonstrated stable individual differences in cognitive performances over time (Bohn *et al*., 2023). The same population was tested every 2 weeks for 18 months on the same cognitive tasks: a gaze‐following task, a task of causal inference, a task of inference by exclusion (identifying the presence of food by sound in a full or empty cup), a quantity discrimination task and a delay of gratification task. Interestingly, individual characteristics (age, group, test experience) were more important than transient events (life events, testing or sociality) to explain task performance. More specifically, for inhibitory control, performances of rhesus macaques in a Go/No‐go task, a modified Stroop task and in a reversal learning task were consistent across periods of 2 weeks (Loyant *et al*., 2022). In another study, performances of passerine birds in successive reversal learning tasks were repeatable (Cauchoix *et al*., 2017). Similarly, Australian magpies (*Gymnorhina tibicen*) tested as juveniles in the cylinder task and reversal learning task repeated their performance as adults (Ashton *et al*., 2018). However, the performance of North Island robins (*Petroica longipes*) in the cylinder task was not repeatable over a year (Shaw, 2017). The choice of response variable may also be a limitation in successfully reproducing inhibitory control performance over time. For example, a continuous variable (such as waiting time) may be more repeatable than a success/failure criterion (e.g. touching a barrier in a detour) as it is less likely to produce binary outcomes. Temporal repeatability could also be affected by confounding factors such as changes in motivation and experience over time. Overall, the field of inhibitory control is still lacking a definitive assessment of the reliability of common inhibitory tasks.

### (3) Floor and ceiling effects

**Fig. 4:**
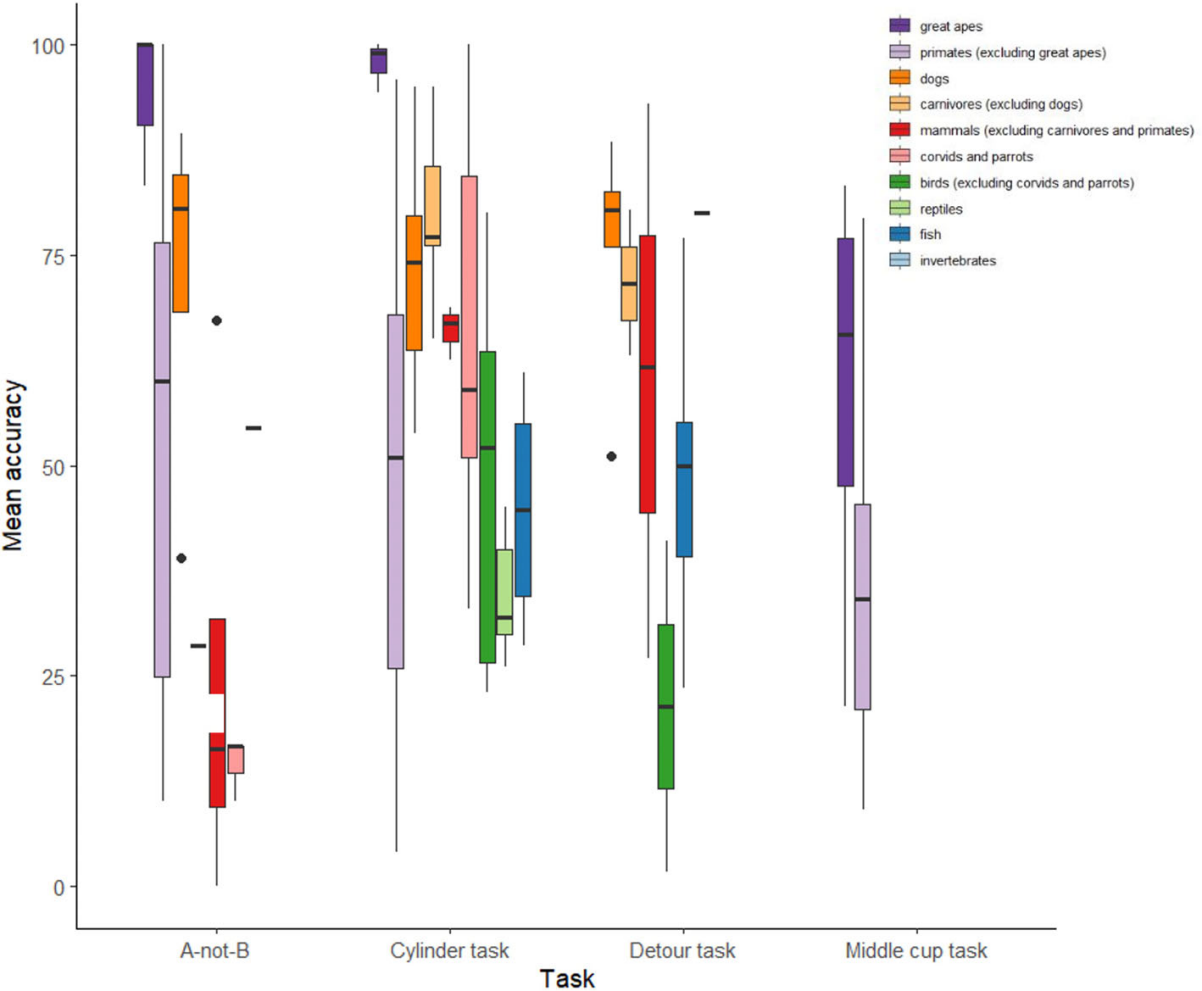
Mean accuracy (% of correct responses) per class of animal in the A‐not‐B task, cylinder task, detour task and middle cup task in our sample of papers (*N* = 318 tests for which task accuracy was recorded). Not all animal classes were tested in all tasks, and only non‐human animals are included. The tasks were carried out with comparable setup and procedure across taxa (see Fig. 1). We chose to represent only tasks included in more than one paper and which were tested in more than one species. The lower and upper hinges correspond to the first and third quartiles (the 25th and 75th percentiles, respectively). The upper whisker extends from the hinge to the largest value no further than 1.5 × interquartile range (IQR). The lower whisker extends from the hinge to the smallest value at most 1.5 × IQR of the hinge. Data beyond the end of the whiskers (outliers) are plotted individually. See Table S3 for detailed results.

**Fig. 5:**
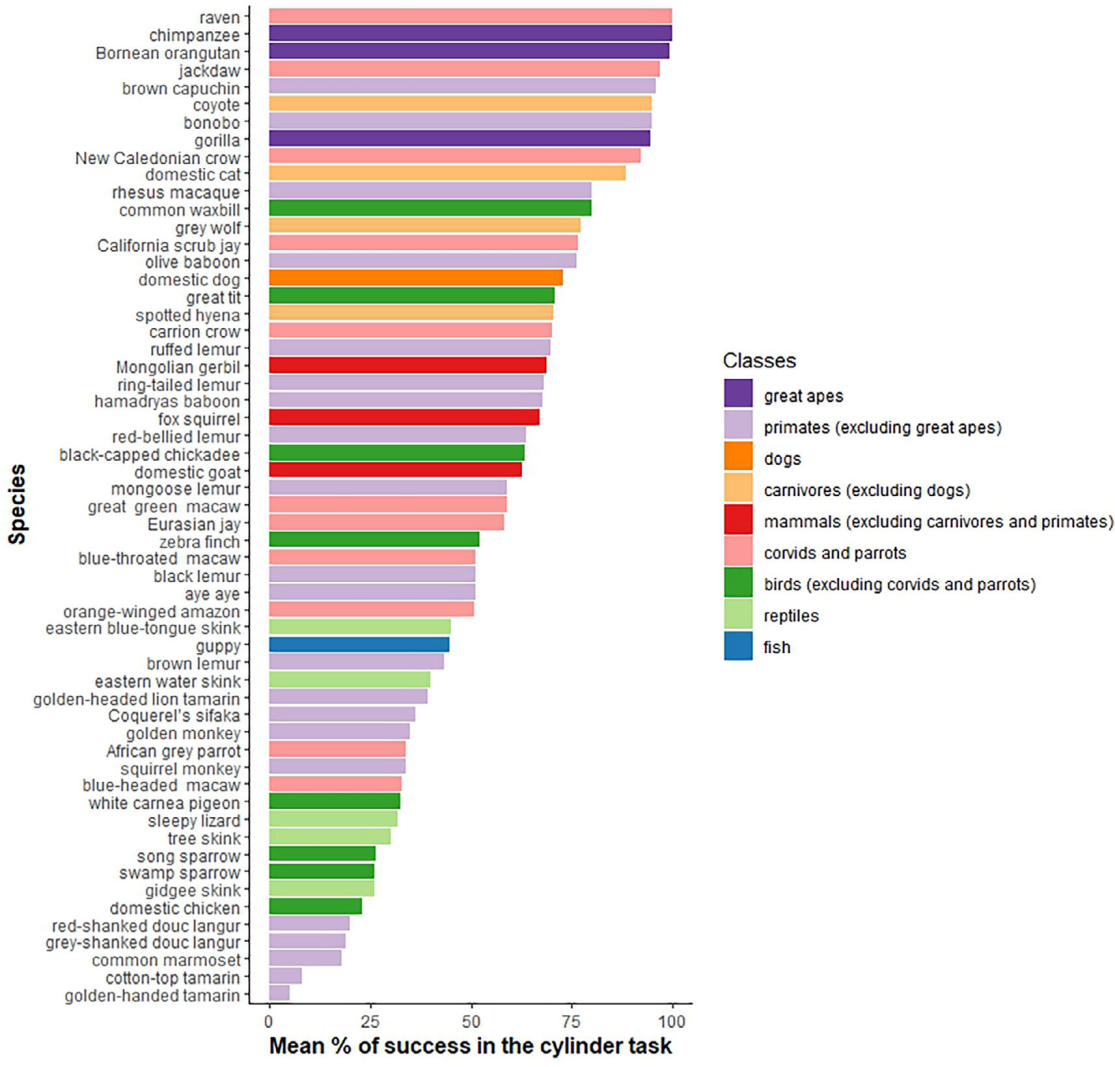
Performance in the cylinder task. Bars represent the mean percentage of correct trials. Error bars were not given for each species so they could not be plotted. The cylinder tasks included were carried out with comparable methods (pre‐training using an opaque cylinder; test experiment using a transparent cylinder).

Floor and ceiling effects, that is when a task is too difficult or too easy, are a common problem in cognitive tasks and are particularly important when comparing inhibitory control across species (Shaw & Schmelz, 2017; Völter *et al*., 2018). These effects can also lead to low variability among subjects, reducing the strength of correlations (Biro & Stamps, 2015; Koo & Li, 2016; Nakagawa & Schielzeth, 2010; Paap & Oliver, 2016). In our sample of papers, the mean accuracy measured for a specific task (% of correct responses) was quite variable depending on the species studied (Figs 4 and 5). Great apes showed the best performances in the A‐not‐B and cylinder tasks, with a mean accuracy close to the ceiling in both tasks (Fig. 4, Table S3). However, great apes had a lower accuracy of 24.19% in the swing door task (Table S3). Primates excluding great apes exhibited worse performances than great apes in all tasks for which comparisons were possible (Fig. 4 and Table S3). In the swing door task, none of the three species (spider monkey, *Ateles geoffroyi*, long‐tailed macaque, *Macaca fascicularis*, brown capuchin, *Cebus apella*) tested succeeded. In the cylinder task (Fig. 5), the performances of primates excluding great apes were surprisingly varied. The second‐best performers overall with a mean accuracy in the cylinder task of 72.87% and 79.79% seem to be the dogs and the carnivores (excluding dogs) respectively (Table S3). Corvids and parrots were tested in only the A‐not‐B and cylinder task, with none succeeding at the former but showing a reasonable performance of 65.62% at the cylinder task. Ravens (*Corvus corax*) had an accuracy of 100% in the cylinder task. Fishes had average performances in the detour task (48.46%). Finally, mammals (excluding carnivores and primates) performed worst at the A‐not‐B task (24.94%) but better in the cylinder task (66.07%). One species, the Asian elephant (*Elephas maximus*) had a mean accuracy of 0 (*N* = 5 elephants; MacLean *et al*., 2014). Overall, there are clear differences among species in task difficulty, potentially hindering the ability to identify meaningful patterns (Völter *et al*., 2018). For the tasks included in Fig. 4, there appear to be few examples of floor or ceiling effects, potentially because researchers use pilot studies to adapt tasks to the morphology, and sensory and cognitive abilities of each species. However, for both the A‐not‐B task and cylinder task, the performance of great apes is close to the ceiling. It may be that this type of task, involving hand tracking and hand manipulation, is more ecologically relevant to social primates that use their hands to forage than it is to other animals that forage with their mouth or beak. Jelbert *et al*. (2016) demonstrated that New Caledonian crows (*Corvus moneduloides*) trained to track rewards moved by a human demonstrator were more likely to succeed at the A‐not‐B test than birds trained on an unrelated choice task involving inhibitory control. A lack of ecological validity of the common inhibitory control tasks could partly explain the variability in results among species (Schubiger, Fichtel & Burkart, 2020).

### (4) Factors influencing inhibitory control

Even when inhibitory control performances are stable within an individual, confounding factors, frequent in animal cognition, can influence performances differently across individuals and tasks (Schubiger *et al*., 2020; see Table S4), making it difficult to obtain a pure measure of inhibitory control within a single task (Völter *et al*., 2018). A large proportion of the observed variation may reflect non‐cognitive or cognitive individual differences or idiosyncratic task requirements with only a small proportion of the variation actually capturing the studied process (Friedman & Miyake, 2017; Gärtner & Strobel, 2019; Jelbert *et al*., 2016; Schubiger *et al*., 2020; Shaw & Schmelz, 2017). This phenomenon, often referred to as the “task impurity” problem, may be inevitable in inhibitory control research (Friedman & Miyake, 2004; Gärtner & Strobel, 2019; Völter *et al*., 2018). For instance, the validity of the cylinder task, one of the benchmark tests in inter‐species comparisons (MacLean *et al*., 2014), is now under debate due to the involvement of confounding factors such as experience, learning, motivation, etc. (for reviews see Kabadayi *et al*., 2016, 2017b; Shaw & Schmelz, 2017).

#### (a) Context of the task

The absence of significant correlations between inhibitory control tasks in the literature is often explained by differences in context between tasks (Bray *et al*., 2014; Fagnani *et al*., 2016; Gatto, Lucon‐Xiccato & Bisazza, 2018; van Horik *et al*., 2019; Vernouillet *et al*., 2018). With respect to inhibitory control, “context” often refers to whether the subject is required to inhibit a social or appetitive impulse. For example, Bray *et al*. (2014) argued that a difference in context explained the absence of correlation in inhibitory control performances of dogs in tasks involving a social or a reward‐based paradigm. Similarly, the inhibitory performances of guppies have been tested in different contexts with a detour task using prey (Lucon‐Xiccato, Bisazza & Bertolucci, 2020a) or social partners (Gatto *et al*., 2018) as a reward. However, the effects of the differences between these two contexts have not yet been tested.

#### (b) Individual factors

##### (i). Individual attributes

Inhibitory control performances can be strongly affected by the individual's characteristics (see Table S4), such as the sex of the animal. This effect is known to be particularly strong in the emotional Stroop task in humans, with men having an attentional bias towards threatening stimuli (Paul, Harding & Mendl, 2005; Sass *et al*., 2010; Smith & Waterman, 2005). Animal studies have reported mixed results. A study in capuchin monkeys found no sex difference in the distractive effect of a threatening stimulus (Boggiani *et al*., 2018). However, Loyant *et al*. (2021) found stronger distraction towards threatening pictures in male rhesus macaques compared to females.

Inhibitory control abilities can also change with ageing (Bedard *et al*., 2010). An impairment in performance with age has been demonstrated in the reversal learning task. Older dogs (Bray *et al*., 2014; Tapp *et al*., 2003; Wallis *et al*., 2016), older rats (Schoenbaum *et al*., 2002) and older non‐human primates (Bonté, Kemp & Fagot, 2014; Kumpan, Smeltzer & Teichroeb, 2020; Tsuchida, Kubo & Kojima, 2002) all showed less flexibility in reversing a previously learned rule.

##### (ii). Training and experience

Training and prior experience with behavioural experiments is a well‐known potential confounding factor for inhibitory control (Duque & Stevens, 2017; Kabadayi *et al*., 2018; van Horik *et al*., 2018b, 2019; Vernouillet *et al*., 2018, see Table S4). Experienced individuals may have a greater physical understanding of the task [e.g. object permanence, spatial relationships, transparency (Duque & Stevens, 2017; Kabadayi *et al*., 2017b, 2018)]. For example, one study demonstrated that New Caledonian crows trained to track rewards moved by a human demonstrator were more likely to pass the A‐not‐B test (Jelbert *et al*., 2016). These trained birds performed at a similar level to non‐human primates, contradicting the findings of MacLean *et al*. (2014). In common pheasants, learning, type of training, and prior experience with transparent surfaces have been demonstrated to improve inhibitory control performances (van Horik *et al*., 2018a, 2019, 2020).

##### (iii). Motivation

Another important factor is the motivation of the subject, which can strongly influence inhibitory control performances (Brucks, Marshall‐Pescini & Range, 2019; Duque & Stevens, 2017; Schubiger *et al*., 2020; Shaw & Schmelz, 2017). For example, motivation can be affected by the visibility or salience of the reward, with weaker response inhibition for more visible rewards (Brucks *et al*., 2017; Kabadayi *et al*., 2017b, 2018). Indeed, one explanation proposed for the disparity in performances in the detour task and in the A‐not‐B tasks is a difference in the salience of the reward (Bray *et al*., 2014; Brucks *et al*., 2017; Fagnani *et al*., 2016; Marshall‐Pescini *et al*., 2015; Vernouillet *et al*., 2018). Motivation could potentially explain differences within a task or between tasks, as it is associated with an individual's internal desire/physiological demands. For instance, a hungrier individual would be more motivated to obtain a food‐related reward and thus less able to exert inhibitory control. *Ad libitum* feeding is often used in animal testing environments to control for this type of bias. Reward type and quantity may also affect comparisons among individuals, cohorts, and species. Hence there is a need for detailed and standardised protocols for interspecies comparisons and for task validity.

##### (iv). Personality

The personality of a subject may also influence their performances in cognitive tasks (Griffin *et al*., 2015; Boogert *et al*., 2018; Schubiger *et al*., 2020, see Table S4). Here we refer to “personality” as stable individual differences in behaviour that are repeatable across time and/or contexts (Réale *et al*., 2007). In humans, it has been demonstrated that anxious people have an attentional bias towards negative stimuli (MacLeod, Mathews & Tata, 1986; Bar‐Haim *et al*., 2007). Non‐human primates that had recently experienced a traumatic experience showed a similar bias towards threatening stimulus (Allritz *et al*., 2016; Bethell *et al*., 2012, 2016; for review see Crump, Arnott & Bethell, 2018). This attentional bias was also linked to fearful temperament in rhesus macaques (Bethell *et al*., 2019). Attentional bias may be relevant to inhibitory control as the animal needs to inhibit a prepotent response (reaction to emotional stimuli) while performing a task. A personality effect on inhibitory control performances has been demonstrated in fish. In both zebrafish (*Danio rerio*) and guppies, bolder individuals showed greater inhibitory control skills when tested in a variant of the cylinder task (Lucon‐Xiccato *et al*., 2020b).

##### (v). Other cognitive processes

Any inhibitory control task involves other cognitive processes such as learning ability, spatial cognition, problem solving, memory, etc. (Diamond, 2013; Kabadayi *et al*., 2018; Shaw & Schmelz, 2017), which makes it difficult to disentangle the processes involved. Differences in learning abilities might explain why performance in tasks measuring a spontaneous response (such as in the modified Stroop task and Go/No‐go task) are not consistent with tasks that involve higher cognitive abilities such as the reversal learning task (Loyant *et al*., 2022). Similarly, a lack of correlation between the detour task or cylinder task and the A‐not‐B task (Bray *et al*., 2014; Brucks *et al*., 2017; Fagnani *et al*., 2016; Marshall‐Pescini *et al*., 2015; Vernouillet *et al*., 2018) could be explained by the involvement of other cognitive processes. van Horik *et al*. (2020) demonstrated that response learning (using reinforcement of motor responses to a transparent barrier) strongly increased performance in the cylinder task in common pheasants.

#### (c) Environmental factors

##### (i). Developmental environment

The environment in which an individual develops is also an important confounding variable in inhibitory control experiments (Schubiger *et al*., 2020). An individual developing in a more stimulating and enriched environment is likely to develop better cognitive skills (e.g. cognitive flexibility). For example, guppies recently derived from a wild population seem to perform better than domesticated guppies at a detour task (Gatto *et al*., 2018). Similarly, shelter dogs displayed poorer performances in the A‐not‐B task than did pet dogs (Fagnani *et al*., 2016). According to the authors, shelter dogs might have less interaction with humans which would decrease their chances of learning to inhibit certain behaviours. Lastly, common pheasants reared in spatially unpredictable environments demonstrated greater inhibitory control ability compared to those reared in predictable environments (van Horik *et al*., 2019).

##### (ii). Social factors

The social environment in which an individual interacts is also a key factor influencing variations in inhibitory control ability (Schubiger *et al*., 2020). The social intelligence hypothesis (Dunbar, 1998; Humphrey, 1976) postulates that the demands associated with a complex social life, with differentiated social relationships, generate selection for increased brain size and higher cognitive performance, such as inhibitory control (e.g. Wascher *et al*., 2018). Group size has been used as a proxy for social complexity (Bergman & Beehner, 2015; Kappeler, 2019). For instance, in spotted hyenas (*Crocuta crocuta*), living in a larger group seems to be associated with better inhibitory control skills (Johnson‐Ulrich & Holekamp, 2020) in a cylinder task. Similar results were found in Australian magpies, using the cylinder task and the reversal learning task (Ashton *et al*., 2018). The rank of an individual might also be an important social factor: lower ranked hyenas living in larger groups seem to have better inhibitory control than higher ranked conspecifics in a cylinder task (Johnson‐Ulrich & Holekamp, 2020). The social environment in which an experiment is conducted also might affect inhibitory control performance. For instance, chimpanzees exhibited greater self‐control when alone than when tested alongside a conspecific (Evans *et al*., 2012). In another study, the presence of a social partner decreased the inhibitory performances of baboons engaged in a reversal learning task (Huguet *et al*., 2014).

### (5) Low sample size

**Fig. 6:**
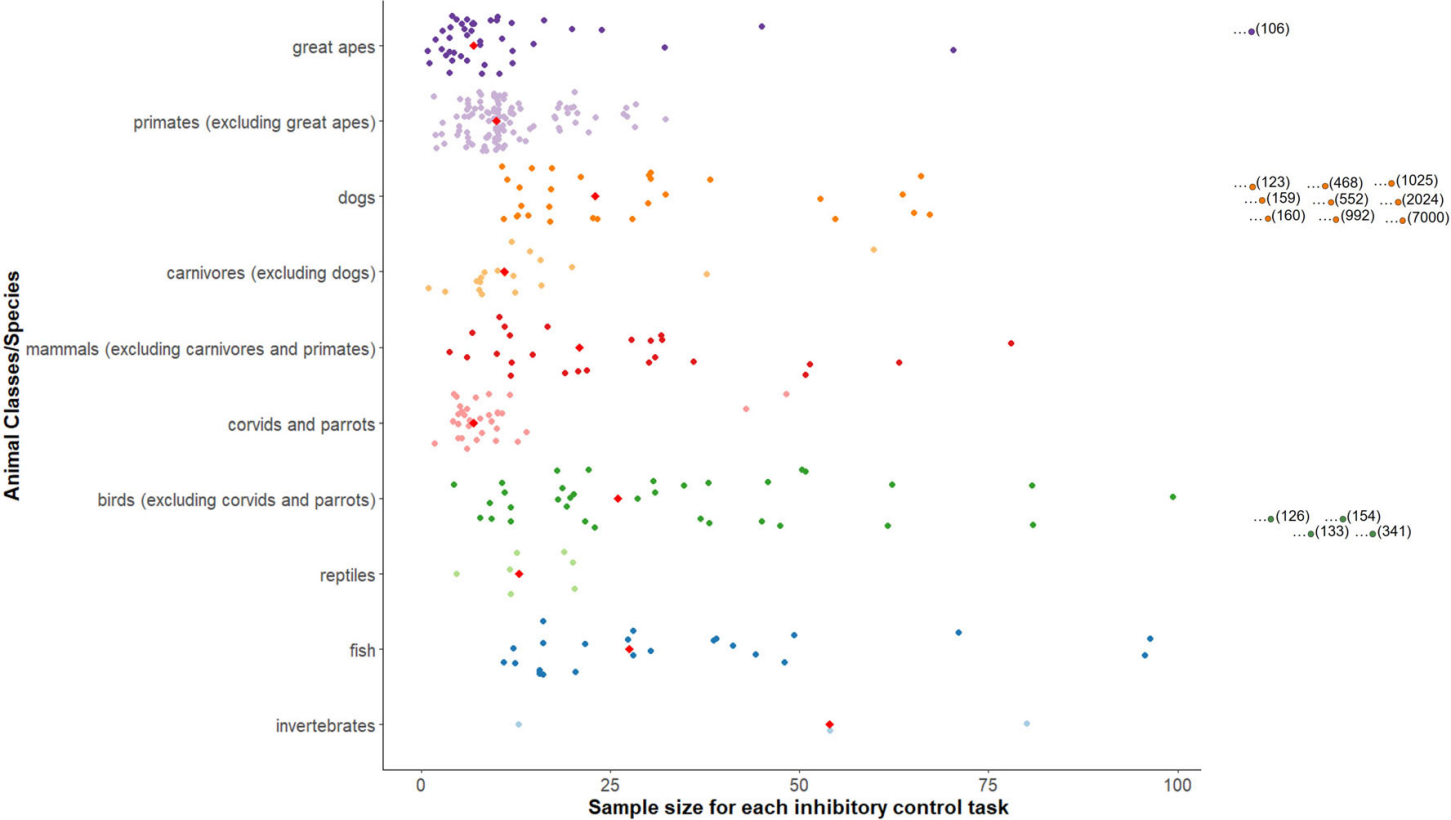
The distribution of sample sizes for inhibitory control tasks used in our sample of papers for different animal taxa (*N* = 339 different sample sizes collected). The red diamond indicates the median. “…” indicates an outlier not included for readability on this scale with its sample size *N* given in parentheses.

Another common challenge in using animals in research is a low sample size, which will decrease the analytical power and could lead to unreliable results (Koo & Li, 2016; Paap & Oliver, 2016; Völter *et al*., 2018). For the inhibitory control studies selected herein, the sample size often is relatively low but with a large range [median = 12; mean ± S.D. (*N*) = 60.22 ± 404.91; see Fig. 6 and Table S5]. The number of subjects in our sample of papers ranged from one chimpanzee (Beran *et al*., 2007) to 7000 dogs (Horschler *et al*., 2019). The lowest median sample sizes were found for studies on corvids and parrots (7) and great apes (7) (Table S5, Fig. 6). The same small median sample size (7) for great apes was found both in a meta‐analysis on animal physical cognition (Farrar *et al*., 2020) and in a meta‐analysis of 574 primate cognition papers (ManyPrimates *et al*., 2019).

The largest median sample sizes were for studies on invertebrates (54), dogs (30) and birds excluding corvids and parrots (31) (Fig. 6 and Table S5). The higher sample sizes in birds are due to large‐scale inhibitory control studies in common pheasants from breeding farms [e.g. Langley *et al*. (2020) reported results from 341 pheasants]. The large numbers of dogs used in some studies can be explained by the accessibility of this species and the recent use of citizen science in dog research (see Hecht & Rice, 2015), which can result in high numbers of participants. Researchers may have only limited access to some species kept in captivity (such as primates and large mammals), and due to more recent moves to reduce the number of subjects used in animal research imposed by the Institutional Animal Care and Use Committee. Captive animals can also experience different rearing conditions, different lifespans for long‐lived species or different prior experience with psychological testing which can affect interpretations of results. Researchers must achieve a balance between ethical and methodological requirements. Unfortunately, small sample sizes can prevent a researcher from detecting effects or lead to the identification of spurious effects (ManyPrimates *et al*., 2019; Shaw & Schmelz, 2017; Völter *et al*., 2018). A small sample size can also limit the ability to generalise from a sample to the entire population (ManyPrimates *et al*., 2019). However, for some relatively under‐researched taxa (dogs, birds, fishes or insects, see Table S5), access to larger sample sizes has enabled studies of inhibitory control.

## IV. FRAMEWORK TO MEASURE INHIBITORY CONTROL

### (1) Development of a task of inhibitory control

**Fig. 7:**
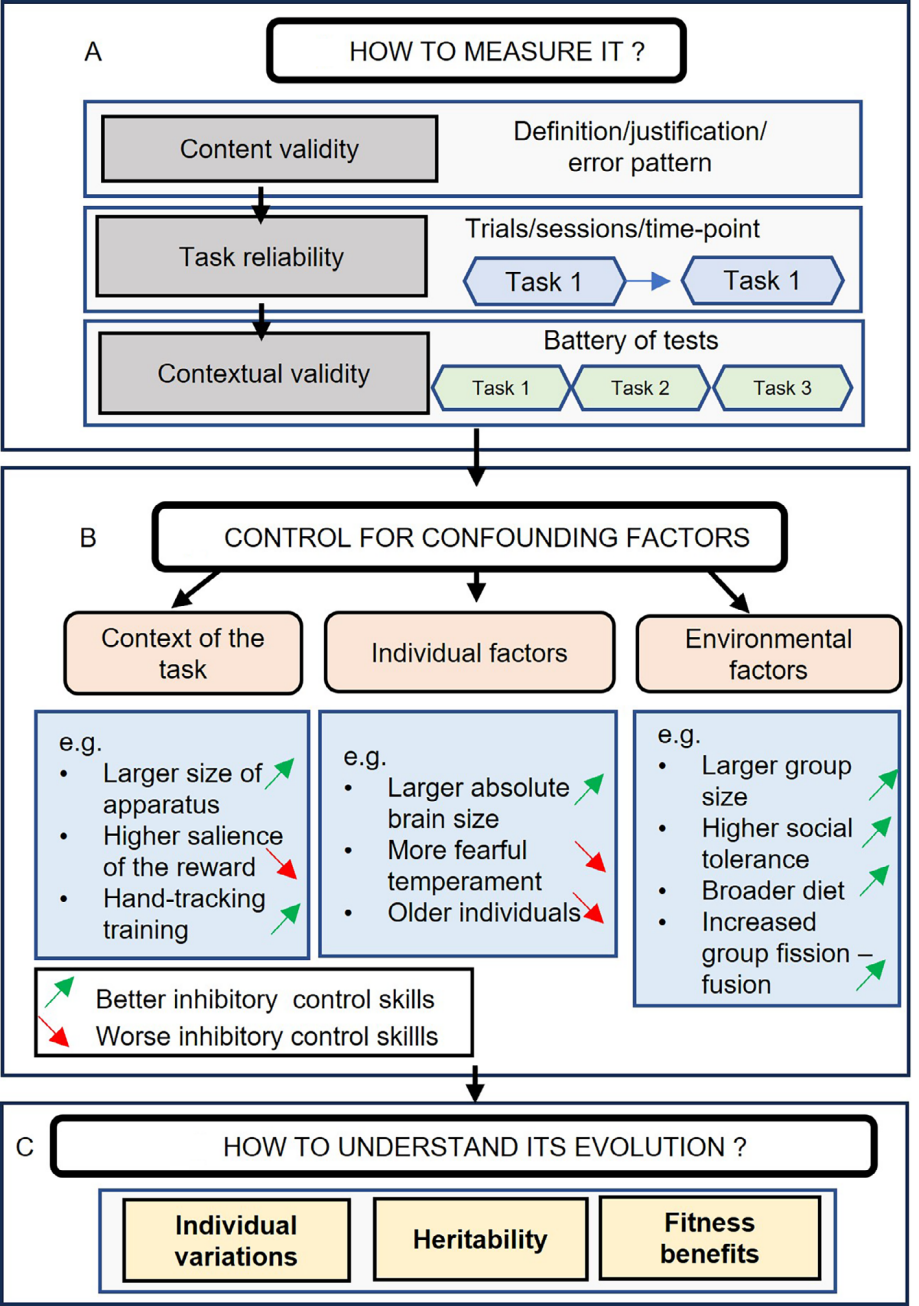
Suggested framework to study inhibitory control in animals. (A) Establish the content validity of a chosen task by clearly defining the ability of interest, justify the choice of the task and demonstrate a characteristic error pattern; then assess the test–retest reliability of this task over trials, sessions and time points; finally, use a battery of tasks to demonstrate the contextual validity of several tasks of inhibitory control putatively measuring this ability. (B) Researchers then should control for individual, environmental and task‐related confounding factors that could influence inhibitory control performances (green arrows represent a positive influence on inhibitory control performances, red arrows a negative influence). (C) To understand better the evolution of inhibitory control, researchers should finally focus on the individual variations, heritability and fitness benefits associated with this trait. See Table S4 for a detailed list of subject‐ and task‐related factors that can influence inhibitory control performance.

In line with the classical psychometric literature, we here provide a framework (see Fig. 7) to develop inhibitory control tasks.

#### (a) Defining the studied ability

The first step in developing a valid task of inhibitory control is to define clearly the ability tested (see Fig. 7A; Friedman & Miyake, 2004; Völter *et al*., 2018). Across different fields of study, the term “inhibition” has been used inconsistently (Beran, 2015; Friedman & Miyake, 2004) and various terminologies have been used to refer to related processes (e.g. self‐control, response inhibition, behavioural inhibition, cognitive control, interference control, self‐regulation inhibition, etc.). This “terminological morass” as described by Beran (2015), could impact efforts to define inhibitory control clearly. We follow Beran (2015) by defining inhibitory control as the ability to control impulsive behaviours (also known as a prepotent response; see Fig. 1) in order to reach a more rewarding goal (i.e. succeeding in a task, or reaching a criterion of success; Fig. 1). We can subdivide inhibitory control into three categories of constructs: (*i*) the inhibition of an impulsive action, where a prepotent automated motor response needs to be inhibited in order to succeed in a task. For example, in the cylinder task an impulsive behaviour (reaching towards a transparent surface) must be inhibited in order to access a reward. (*ii*) The inhibition of a cognitive set, where a pre‐learned rule must be inhibited in order to succeed (e.g. the A‐not‐B task). (*iii*) The inhibition of a response to an internal (an emotional reaction) or an external stimulus in order to complete a goal‐oriented task. Unlike inhibitory control tasks where the subject is actively performing a task, in a self‐control task, the subject needs to wait passively for the goal outcome (e.g. in inter‐temporal choice or delay‐of‐gratification tasks). Due to variable terminology, it is important always to define the prepotent response that is inhibited, the category of the task, and the correct response. For example, a study of inhibitory control in Tonkean macaques (*Macaca tonkeana*) using the A‐not‐B task should state that they are studying the inhibition of a pre‐learned set (cognitive set inhibition), the prepotent response is reaching for a previously rewarded location, and the correct response is reaching for the newly rewarded cup.

#### (b) Choice of the task

In the literature, there is often little justification of the selection and content validity of a task (defined as the degree to which a measurement is representative of the targeted construct) putatively measuring inhibitory control (Friedman & Miyake, 2004; Völter *et al*., 2018). According to Völter *et al*. (2018), two main approaches can guide the selection of a cognitive task: an “ethological approach” and a “psychological approach”. Using the ethological approach requires that a task is relevant to a species' socio‐ecological challenges, and thus the use of fitness‐related abilities. For example, the cylinder task assesses the problem‐solving ability of an individual to locate food resources, while the emotional Stroop task tests the social ability to process a threatening cue. In the psychological approach, an experiment is devised on the basis of the existing literature, for example from other species, to produce oriented hypotheses. In human research, knowledge on the neurobiological underpinnings of inhibitory control is more advanced due to evidence from neuropathologies associated with a lack of response inhibition (such as in attention deficit hyperactivity disorder and addictions; Aron & Poldrack, 2005). However, the application of established tasks to a new species may require adjustments through, for instance, pilot studies. For example, the cylinder task for mammals was adapted for fish by using a glass test tube (Lucon‐Xiccato *et al*., 2020a). According to the ethological approach, the authors would have to justify that reaching for prey in a glass tube is a reasonable approximation of a natural ecological challenge. Any extrapolation of experiments to a new species thus requires careful assessment of its sensory system and motor limitations. For example, when planning a study of inhibitory control in Tonkean macaques using the A‐not‐B task, researchers should justify the ethological relevance of this task (e.g. the behavioural flexibility of this species while foraging for insects under rocks), the choice of the apparatus (for example, the choice of cup size or colour, are they easily discriminated and manipulated by macaques?) and procedure (e.g. what is the attention span of this species?), with reference to previous research in this species or on other species of primate (psychological approach).

#### (c) Characteristic error pattern

Demonstrating a characteristic error pattern that reflects individual variation in performances (i.e. a signature limit of performance) can help to assess the content validity of a task (Hedge, Powell & Summer, 2018; Völter *et al*., 2018; Fig. 7A). For an inhibitory control task, a specific error pattern indicates that a prepotent response has been overridden in order to complete the task successfully. Such an error pattern should be present in all individuals but errors are likely to be more frequent in inexperienced animals or in animals with a lack of inhibitory control, for example due to differences in rearing environment, personality, sex, etc.). For instance, in the Go/No‐go task, some individuals may fail to withhold their prepotent response to touch the No‐go stimulus. To assess the error pattern, subjects need to be tested in a sufficient number of trials. However, using too many trials would introduce opportunities for learning which could confound testing of inhibitory control (van Horik *et al*., 2020), while in binary choice tests (e.g. reversal learning) there must be sufficient trials to exclude random choices. Researchers can also manipulate the complexity of the task by increasing the strength of the dominant response. For instance, the salience or value of the reward can be enhanced, or researchers can increase use of the Go stimulus in the Go/No‐go task. At the group level, there must be systematic variation across conditions (e.g. between the Go and the No‐go stimulus). To rule out the possibility of random responses, irrelevant stimuli can be introduced.

In our example with Tonkean macaques using the A‐not‐B task, after the reversal phase (in which the reward location has been changed), the characteristic error pattern could be assessed as the frequency of attempts to access the reward at the first rewarded location. To exclude random responses researchers can analyse the response to a third cup which has never been baited to distinguish between inhibitory control errors (reaching for the first cup baited) and unspecific mistakes (Völter *et al*., 2018).

### (2) Reliability

Before considering using an inhibitory control task researchers need to assess its reliability (Griffin *et al*., 2015; Miyake *et al*., 2000; Völter *et al*., 2018; see Fig. 7A). There should be consistent individual performances between trials, sessions and time points (i.e. repeatability) and consistent rankings among group members. For instance, when determining the interval between two time points for testing, the interval between these time points should neither be too long to avoid dramatic changes in the internal and external states of the subjects (see Bell, Hankison & Laskowski, 2009; Shaw & Schmelz, 2017) nor too short to avoid the influence of carryover effects such as arousal or short‐term memory (Bell *et al*., 2009). In humans, repeatability of inhibitory control tasks used intervals ranging from 9 to 28 days (Wöstmann *et al*., 2013; Weafer *et al*., 2013). In a meta‐analysis of the repeatability of behaviour Bell *et al*. (2009) demonstrated that individuals are more consistent over shorter intervals (less than a year) compared to longer intervals (more than a year). Repeatability of behavioural tests has been assessed at two‐week intervals in non‐human primates (Bohn *et al*., 2023; Uher, Asendorpf & Call, 2008; Loyant *et al*., 2022). Unfortunately, to the best of our knowledge, there is no specific justification of the most appropriate length for the period between tests in the literature. Using our previous example, after initial testing of Tonkean macaques in the acquisition and reversal phase, they should be tested again after a defined period (usually 2 weeks for studies on primates, but this interval would need justification) using a new reversal experiment (e.g. from the previously reversed rule to a new reversed rule).

### (3) Contextual validity

A cognitive task is never a pure assessment of one cognitive process. For example, the reversal learning task putatively requires inhibition of a pre‐learned rule but also involves other cognitive processes such as memory or attention (Diamond, 2013; Kabadayi *et al*., 2018; Shaw & Schmelz, 2017). To control for the involvement of other non‐targeted cognitive processes (also known as the “task impurity problem”) and to reveal a common underlying cognitive construct, we recommend (Fig. 7A) use of a battery of valid and reliable tasks (Beran, 2018; Cauchoix *et al*., 2018; Friedman & Miyake, 2017; Griffin *et al*., 2015; Shaw & Schmelz, 2017; Völter *et al*., 2018). A battery of tasks can enable extraction of the common underlying processes and mitigate the variance introduced by confounding factors, reducing the problem that correlations or experimental effects may be due to non‐inhibition‐related variance [i.e. convergent validity (Friedman & Miyake, 2004; Völter *et al*., 2018)]. A battery of inhibitory control tasks should demonstrate cross‐consistency in an individual's performance and convergence on a single result (Griffin *et al*., 2015; Shaw & Schmelz, 2017). In our worked example, Tonkean macaques could be tested using several tasks of inhibitory control (e.g. Go/No‐go task and a Stroop task) to investigate consistency in performance. In common pheasants, van Horik *et al*. (2018b) tested their subjects with nine cognitive tasks including two inhibitory control tasks. They found no evidence to suggest that a single factor encompassed these cognitive abilities. Conversely, tasks that aim to measure different constructs would be expected to show divergent validity or discriminant validity (Völter *et al*., 2018). A deeper problem would be if the different included tasks share a common process other than inhibitory control, that is whether there is shared variance due to inhibitory control ability or from other irrelevant factors. For example, attention is required in the Go/No go task, the Stroop task and the reversal learning task. If a subject has a low level of attention, it could show low performances on these tasks independently of its inhibitory control performances. To avoid such issues, researchers could choose tasks that differ as much as possible in peripheral task demands.

A potential solution to minimise the task impurity problem and to control for other constructs would be to include “micro‐behaviour” (Cauchoix *et al*., 2018). For example, Chow *et al*. (2017) tested eastern grey squirrels (*Sciurus carolinensis*) in a colour discrimination–reversal learning task, and analysed the number of errors to learn the rules and the response latencies to correct and incorrect stimuli together with the rate of head‐turning as a measure of attention. Another solution would be to include a direct measurement of another influencing construct, for example a memory task. In this way, researchers could compare performances in the inhibitory control tasks (which also requires memory) and the memory task to try to disentangle the different constructs involved. However, memory tasks also rely on attention or learning, as do inhibitory control tasks, and it remains difficult to isolate each construct statistically. Finally, it would be of interest to develop a task that combined both memory ability (e.g. a match‐to‐sample task), and inhibitory control ability (e.g. by including distractors as stimuli in the match‐to‐sample task). Researchers could then analyse if performances in this hybrid task were consistent with a pure memory task or a modified Stroop task. van Horik *et al*. (2020) demonstrated in common pheasants that reinforcement of a fixed motor response (as opposed to a changing motor response) improved inhibitory control performances in the cylinder task, suggesting that response learning can confound assays of inhibitory control.

### (4) Control for confounding factors

The next important challenge is to minimise the impact of any confounding factors (e.g. non‐cognitive or cognitive factors) that could influence the test results independently of the studied process (Fig. 7B).

#### (a) Wild versus captivity

Potential confounding factors can be controlled for in a balanced and randomised test design. The population tested should include a representative sample of individuals (for example, the same number of males and females, a wide age range, etc.). In animal research, most of our present understanding of animal cognition originates from research conducted in laboratories, sanctuaries and zoos, which can limit the diversity and the choice of subjects. While experiments conducted with captive animals can allow control of intrinsic and extrinsic factors that can influence animal behaviour (e.g. test context, developmental factors, experience), the sample tested might be representative only of one group of individuals from one population in one specific context, limiting our ability to extrapolate the results to wild animals. In addition, research with captive animals removes animals from their natural settings and may lead to abnormal behaviours (stereotypical behaviours) or unusual ecological conditions (small territories or group size) which can affect their performances. Some research teams recently have made advances in testing inhibitory control in wild animals. For instance, a reversal learning task was successfully adapted to test wild vervet monkeys (*Chlorocebus pygerythrus*; Kumpan *et al*., 2020); inhibitory control skills have been investigated in more than 60 wild spotted hyenas using a cylinder task (Johnson‐Ulrich & Holekamp, 2020); and inhibitory control skills were assessed in wild Australian magpies (56 adults and 21 juveniles) using one task (Ashton *et al*., 2018). A field‐based approach is complementary to laboratory‐based studies and enables assessment of whether cognitive abilities found in captive subjects are generalizable to wild animals (see Cauchoix *et al*., 2017). However, there remain major problems associated with using wild animals, including experience, motivation, social context etc., as we know that these can affect inhibitory control (van Horik *et al*., 2018a, 2019; Kabadayi *et al*., 2018; Jelbert *et al*., 2016).

#### (b) Minimising or measuring sources of variation

When designing an experiment, factors influencing the variability among individuals or groups can either be studied (i.e. when researchers focus on individual differences), minimised (i.e. subjects should be raised and kept in the same conditions), or controlled for statistically during analysis. For example, if participation in an experiment is voluntary, only the most motivated or the tamest individuals may be willing to interact with the experimental setup (Schubiger *et al*., 2020). To control for such variability, subjects can, for example, be fed *ad libitum* (to minimise variability in food motivation) or habituated to the experimental setup (to minimise impacts of anxiety level). However, some personality traits (e.g. extraversion or sociability) cannot be directly controlled for in this way. Researchers can assess these personality traits before training, using standardised procedures, and include this in analyses (e.g. Madden *et al*., 2018; Lucon‐Xiccato *et al*., 2020b). Researchers can for instance, estimate the influence of perseverance by measuring the number of contacts with the wrong option. Similarly, facial expressions or body scratches could be used to assess anxiety levels. In our example study of inhibitory control performance in Tonkean macaques, researchers could feed the tested group *ad libitum* to minimise motivation bias, habituate them beforehand to the apparatus (to minimise anxiety), and test animals only with close associates present (to minimise the stress of being separated from others or being attacked).

#### (c) Choosing control variables

**Fig. 8:**
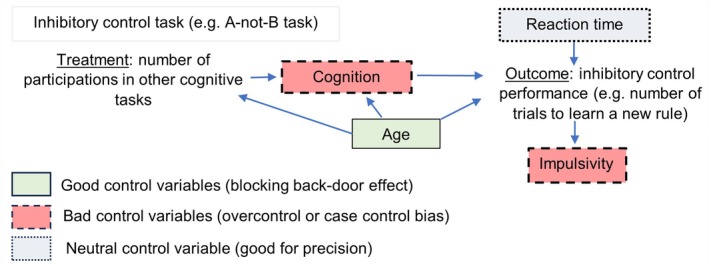
Hypothetical directed acyclic graph illustrating impacts of good (e.g. age of the subjects), neutral (e.g. reaction time of the subjects) and bad control variables (e.g. cognitive performances of or impulsivity of the subjects) on the effect of past experiences on inhibitory control performances in the A‐not‐B task. Arrows represent possible direct causal effects between a variable and an outcome. Blocking back‐door effect means controlling for a variable that is a cause of both the treatment and the outcome (here “age”). Overcontrol is controlling a variable on a causal path between treatment and outcome (here “cognition”). Case control bias is controlling for a variable which is derived from the outcome variable (here “impulsivity”).

Adding too many control variables can make it difficult to make reliable inferences (see Cinelli, Forney & Pearl, 2022). To illustrate this, in Fig. 8 we use our example of a study of inhibitory control using the A‐not‐B task in Tonkean macaques. In this example, we are putatively testing the effect of the number of times an individual has been tested in other cognitive tasks (the treatment variable) on inhibitory control performance (the outcome variable, here the number of trials needed to learn the reversed rule). Arrows in Fig. 8 represent possible direct causal effects between a variable and an outcome, but the effects of the selected variables are hypothetical.

According to Cinelli *et al*. (2022) the variable “age” can be considered as a good control as it is a common cause (confounder) of both past experiences (as individuals age they have more chance to have participated in more cognitive tasks) and inhibitory control performances. Indeed, “age” has been negatively associated with performance in the A‐not‐B task in macaques (Loyant *et al*., 2023). This variable could thus produce non‐causal associations between both the number of participations in cognitive tasks and the inhibitory performances The backdoor adjustment method (i.e. blocking back‐door paths) involves controlling for all variables (here the variable “age”) that are causes of both treatment and outcome. This method involves blocking the association between the treatment (“number of participations in other cognitive tasks”) and the outcome (“inhibitory control performance”) through controlling for a confounding variable (“age”). In Fig. 8, “reaction time” could be considered as a neutral control as it is not a confounding variable, although controlling for it could reduce variation in the outcome variable and thus help to improve precision of the estimate. “Cognition” can be considered as a mediator variable between past experiences and the measurement of inhibitory control. This should not be controlled as this will block the causal path between the variable of interest and the outcome variable (termed “overcontrol bias”), which can lead to loss of information on the effect of the treatment on the outcome. Finally, “impulsivity” can be considered as derived from the outcome variable, and controlling for it could be equivalent to partially controlling for inhibitory control (case control bias).

### (5) Study of the evolution of inhibitory control

A final step (Fig. 7C) is to investigate potential factors affecting the evolution of inhibitory control. Studies of the evolution of cognition have recently focused on the “Darwinian Holy Trinity” (Thornton & Wilson, 2015), that is natural selection acts upon individual variation in a trait that is heritable and linked to fitness (see Thornton & Lukas, 2012; Thornton & Wilson, 2015; Boogert *et al*., 2018).

#### (a) Individual variation

The first condition for a cognitive ability to be evolutionarily selected is individual variation in this trait. As Darwin (1859, p. 50) pointed out, individual differences are of critical importance in biology, as they “afford materials for natural selection to act on”. While in human psychology, individual differences have been studied for more than 100 years (for review see Sackett *et al*., 2017), in non‐human animal research, individual differences seem to have received very little attention. These variations are often considered as sources of undesirable variability – “noise” – that mask between‐group differences (Griffin *et al*., 2015; Thornton & Lukas, 2012; Shaw & Schmelz, 2017). Performances of individuals are often pooled together, implying that performance a sample of a population is representative of the whole species (Boogert *et al*., 2018; Herrmann *et al*., 2010; Shaw & Schmelz, 2017; Thornton & Lukas, 2012; Völter *et al*., 2018). However, there has been a recent focus on intra‐ and inter‐individual variation in cognitive abilities (for review see Boogert *et al*., 2018; Shaw & Schmelz, 2017; Völter *et al*., 2018; Thornton & Lukas, 2012; Wascher *et al*., 2018). According to these authors, investigating individual variation in cognition within a species could shed light on the causes and consequences of individual differences and will allow more informative comparisons across species. Even though several studies have argued for an emphasis on individual differences in animal cognition (Boogert *et al*., 2018; Herrmann *et al*., 2010; Loyant *et al*., 2022; Sauce & Matzel, 2013; Thornton & Lukas, 2012), few have focused on individual differences in inhibitory control. Of these, a study of response inhibition in common pheasants demonstrated large inter‐individual differences in performance (Meier *et al*., 2017), whereas van Horik *et al*. (2018a) failed to identify stable individual variation in the same species using common tasks of inhibitory control. A study in fishes reported individual variations in one detour task (Lucon‐Xiccato *et al*., 2020a). The use of a battery of inhibitory control tasks should enable a better understanding of variability among individual performances.

#### (b) Heritability

Heritability is the second condition for evolution to act upon inhibitory control (Thornton & Lukas, 2012; Thornton & Wilson, 2015). Heritability estimates provide a measure of the relative contribution of genetic and environmental factors in generating phenotypic variation (Croston *et al*., 2015), that is the part of the total variability of inhibitory control that is caused by genetic differences among animals. In humans, inhibitory control is reported to be highly heritable. For instance, Friedman *et al*. (2008) reported that inhibition of a dominant response is highly heritable (*h* ^2^ = 0.99). In another study, Schachar *et al*. (2010) reported moderate heritability (*h* ^2^ = 0.27–0.50) for performance in a modified Go/No‐go task, in more than 900 human families. For animals, we know little about the heritability of inhibitory control performances. In a study of 450 common pheasants across four generations, Langley *et al*. (2020) demonstrated a low to moderate heritability for inhibitory control (*h* ^2^ = 0.17). Use of a validated and reliable battery of inhibitory control tasks to compare performances across generations could enable a better understanding of the heritability of inhibitory control. However, this would require very large numbers of individuals with a well‐resolved pedigree.

#### (c) Fitness benefits

The third condition for natural selection is that the possession of a trait confers a fitness benefit, that is a competitive advantage in terms of access to mating opportunities or resources (Thornton & Lukas, 2012). Given that efficient inhibitory capacities could allow flexibility in hazardous situations (Estep *et al*., 1988; Lindsay *et al*., 1976), it is tempting to propose that inhibitory control skills are indeed positively associated with fitness benefits. However, research findings are contradictory. Performance on a battery of cognitive tasks (including the detour task and the reversal learning task) seem to be associated with greater female reproductive success in wild Australian magpies (Ashton *et al*., 2018). Similarly, performance of male song sparrows (*Melospiza melodia*) in inhibitory control tasks (e.g. a detour task and a reversal task) was correlated with song repertoire size, a trait predictive of reproductive success in this species (Boogert *et al*., 2011). Detour reaching performance was also influenced by body condition in North Island robins (Shaw, 2017). By contrast, the performances of common pheasants in the reversal learning task were negatively related to survival following release into the wild (Madden *et al*., 2018). A battery of inhibitory control tasks could enhance understanding of the relationship between inhibitory control performances and fitness traits (e.g. reproductive success, survival). Additional studies are needed to investigate the fitness consequences of inhibitory control, which again will require tracking large numbers of individuals to assess fitness outcomes reliably.

## V. INTERSPECIES COMPARATIVE STUDIES

We know little about the selective forces that favour the evolution of inhibitory control in animals. Comparative studies often contrast the roles of social *versus* ecological selective factors (Amici *et al*., 2008; MacLean *et al*., 2014) but their findings can be contradictory (Powell, Isler & Barton, 2017; DeCasien, Williams & Higham, 2017).

### (1) Brain size hypothesis

Despite decades of research, the hypothesis that brain size predicts cognitive abilities remains highly controversial (Barrett, Henzi & Rendall, 2007; Powell *et al*., 2017). A meta‐analysis (Reader & Laland, 2002) demonstrated that brain size is correlated with tool use, social learning, and innovation in both mammals and birds. An interspecies comparison of 39 mammalian carnivore species demonstrated that relative brain size predicted problem‐solving success (Benson‐Amram *et al*., 2016). For inhibitory control performances, larger‐brained dog breeds performed better in a task of inhibition of action (Horschler *et al*., 2019). Similarly, in a large interspecies comparison, researchers found a positive correlation between inhibitory control performances and absolute brain volume for primate species (MacLean *et al*., 2014). In Table S6 we compare published data on endocranial volume (ECV) for our range of taxa. Other than elephants, the largest ECV for our sample is found in the great apes, and these species also tend to perform best in inhibitory control tasks (see Fig. 4). However, Asian elephants, which had the highest absolute ECV never succeeded in learning the A‐not‐B task, and several corvid species have a low absolute ECV but perform at a similar level to great apes at this task (Jelbert *et al*., 2016) and the cylinder task (Kabadayi *et al*., 2016; Fig. 5). A better comparative measure may be the relative ECV (= ECV/body mass) (Table S6), although this is not correlated with inhibitory performances (MacLean *et al*., 2014). The validity of both measurements is still much debated (Benson‐Amram *et al*., 2016; MacLean *et al*., 2014; Horschler *et al*., 2019; Reader & Laland, 2002). Using both historical and a new data set, Wartel, Lindenfors & Lind (2019) demonstrated that the choice of included variables fundamentally affected the conclusions as to what drives primate brain evolution. Healy & Rowe (2007) point out substantial problems present in comparative studies of brain size and the lack of a coherent scientific framework. Their concerns include problems with the definition of brain size, data collection (various methodologies have been used to measure brain size), and data analysis (correlations of single brain regions with single complex behaviours).

### (2) Ecological intelligence hypothesis

According to the “ecological intelligence hypothesis” (Milton, 1981), large brain size evolved to allow species to adjust their behaviour adaptively in response to foraging challenges. In this ecological context, inhibitory control would allow animals to inhibit inappropriate motor responses (e.g. maintaining restraint while approaching a prey for ambush predators) and updating behavioural strategies (e.g. exploiting new dietary resources, developing new methods of food acquisition or moving from an unrewarding food patch; Rosati, 2017). In one meta‐analysis, brain size was associated with some ecological variables (home range size, diet and activity period) but not with the size of the social group (Powell *et al*., 2017). For inhibitory control, a large comparative study, using results from the A‐not‐B task and the cylinder task for 567 individuals from 36 species, found a positive association between inhibitory control performances and dietary diversity, particularly in non‐human primates, independently of the size of the social group (MacLean *et al*., 2014). By contrast, common pheasants with poor performance on variants of the detour task and the cylinder task displayed wider dietary breadth (van Horik *et al*., 2018a).

### (3) Social intelligence hypothesis

The social intelligence hypothesis (Dunbar & Shultz, 2007; Humphrey, 1976), postulates that the demands associated with a complex social life, with differentiated social relationships, generates selection for increased brain size and enhanced cognitive performances, such as inhibitory control (Wascher *et al*., 2018). Inhibitory abilities are crucial for forming and maintaining stable social relationships, particularly in complex social conditions (Amici *et al*., 2008, 2018; Aureli & Schino, 2019; Byrne & Bates, 2007; Wascher *et al*., 2018). Individuals need to employ inhibitory strategies in remembering, tracking and managing relationships as they monitor and engage in social events around them (Amici *et al*., 2008; Dunbar & Shultz, 2007). For example, a less‐dominant individual should inhibit reaching for food in front of a more dominant one. Animals may tune their social behaviours in relation to the identity of the social partner with whom they are interacting (Amici *et al*., 2008, 2018; Aureli & Schino, 2019; Byrne & Bates, 2007; Wascher *et al*., 2018). Skills such as cooperation or tactical deception requiring a high level of inhibitory control are particularly adaptive in societies where dominance hierarchies determine access to food and mates (Amici *et al*., 2008, 2018; Byrne & Bates, 2007). For example, in cooperative hunting, an isolated individual would need to inhibit attacking a dangerous prey until the rest of the group arrive.

Several authors have attempted to refine the concept of social complexity (for review see Kappeler, 2019) for instance, in terms of group dynamics (Amici *et al*., 2008), the diversity of differentiated relationships (Fischer *et al*., 2017; Bergman & Beehner, 2015) or group size (Ashton *et al*., 2018; Kappeler, 2019). In a more complex society, an individual would need better cognitive abilities to track and update social relationships (Bergman & Beehner, 2015).

#### (a) Size of the group

The size of a social group has been used as a proxy for social complexity (see Kappeler, 2019). Larger groups may be more cognitively demanding due to an increase in the numbers of differentiated relationships and interactions between groupmates (Bergman & Beehner, 2015; Kappeler, 2019). Performance in the cylinder task in spotted hyenas suggested that developing in a larger group generates better inhibitory control skills (Johnson‐Ulrich & Holekamp, 2020; see Fig. 7B). A similar result was found in Australian magpies using the cylinder task and the reversal learning task (Ashton *et al*., 2018). However, for six species of primates, MacLean *et al*. (2013) did not find an effect of group size on performance in the cylinder task.

#### (b) Group dynamics

Frequently splitting and merging subgroups of variable composition (fission–fusion dynamics) is one aspect of primate social complexity (Amici *et al*., 2008; Aureli *et al*., 2008). A comparative study presented five tasks putatively measuring inhibitory control (the A‐not B task, two variants of the detour task, a middle cup task and a measure of self‐control) to seven species of non‐human primates (Amici *et al*., 2008). The authors found an association between performances on these tasks and the social structure of these species. Species living in more dynamic and fluid social environments (fission–fusion societies) outperformed those with more cohesive group structures (see Fig. 7B). The authors concluded that primates living in more complex social groups require inhibition of inappropriate prepotent responses in a dynamic social environment, and this partly explains why they performed better in detour tasks. In another study in six species of non‐human primates that used tasks of social inhibition and behavioural flexibility, Amici *et al*. (2018) demonstrated inter‐species differences which reflected differences in fission–fusion dynamics. Orangutans and chimpanzees (which have a higher degree of fission–fusion dynamics) showed the highest level of inhibitory skills, while gorillas and capuchin monkeys (more cohesive groups) showed the lowest. The authors argued that species with higher spatiotemporal variation in subgroup size and composition could require higher inhibition to enable them to assess new social situations after fusion events.

#### (c) Social tolerance

Finally, it has been suggested that the organisation of the social environment is an important factor for the evolution of socio‐cognitive skills (Byrne, 1996; Byrne & Bates, 2007). The Machiavellian intelligence hypothesis suggests that in a despotic society, social manipulation and deception would lead to the development of richer socio‐cognitive skills, such as inhibitory control (Byrne, 1996; Byrne & Bates, 2007). An individual living in a more competitive social environment would need to inhibit inappropriate behaviours, such as feeding or mating, in the presence of higher ranked conspecifics (Byrne, 1996; Byrne & Bates, 2007). Conversely, it has also been suggested that an individual living in a more tolerant social context, with more relaxed relationships, more cooperative and affiliative behaviours and fewer conflicts might experience more diverse and complex social interactions (Thierry, 2000). A recent study demonstrated that the complexity of a social system (calculated from indices of social diversity, individual behavioural variations and patterns of interactions between individuals) increases with the degree of tolerance among macaques (Rebout *et al*., 2021). Species characterised by higher social tolerance would therefore develop more diverse relationships, and would need to employ better cognitive skills such as inhibitory control (Bergman & Beehner, 2015; Fischer *et al*., 2017; Powell *et al*., 2017; Wascher *et al*., 2018). Joly *et al*. (2017) demonstrated that more tolerant macaque species outperformed less‐tolerant species in one task of inhibitory control, the middle cup task. Similarly, Loyant *et al*. (2023) tested three macaque species with different degrees of social tolerance in a battery of validated inhibitory control tasks (using the Go/No‐go task, a distraction task with pictures and a reversal learning task). The temporal repeatability and the contextual validity of these tasks were assessed in a previous study (Loyant *et al*., 2022). Loyant *et al*. (2023) demonstrated that higher social tolerance was associated with enhanced inhibitory control performances (see Fig. 7B), with more tolerant species being less impulsive and less distracted by pictures of unknown conspecifics.

## VI. CONCLUSIONS

(1) Inhibitory control is defined as the ability to control impulsive or pre‐learned behaviours in order to reach a more rewarding goal. This ability has been studied in a broad range of fields (developmental psychology, psychopathology, ethology, neuroscience, etc.) and in almost all classes of animals. Yet, despite its importance, the structure of inhibitory control is still debated. Rather than representing a single common construct, authors now consider inhibitory control to be a family of independent subcomponents (Friedman & Miyake, 2004, 2017; Nigg, 2000, 2017).
(2) Animal cognition studies often use tasks adapted and simplified from human research, putatively to measure inhibitory control without justifying the choice of the task. Methodological issues, low sample size and lack of reliability and validity of the different inhibitory control tasks have potentially led to contradictory results and a lack of correlation between different measurements. Developing inhibitory control tasks is challenging because of the importance and variety of individual and environmental factors that may influence an individual's performance (see Table S4).
(3) We herein provide a framework (Fig. 7) to develop inhibitory control tasks by first ensuring the content validity of a task and assessing its reliability. Development of a battery of inhibitory control tasks can be a powerful tool to try to control for the task impurity problem common in such tasks. Our framework is intended to improve the accuracy, repeatability and generalisability of inhibitory control measures.
(4) The development of reliable and valid tasks of inhibitory control will enable a better understanding of the conditions necessary for the evolution of inhibitory control (see Fig. 7). Interspecies comparisons will be essential to provide insights on the selective pressures involved, and whether they are social or ecological.

## Supporting information


**Data S1.** Supplementary Information.
**Table S3.** Accuracy (% correct responses out of the total number of trials) for different tasks of inhibitory control, across different animal taxa.
**Table S4.** Subject‐ and task‐related factors that can influence inhibitory control performance.
**Table S5.** Mean, median and standard deviation (S.D.) number of subjects in tests (*N*) for which the sample size was recorded.
**Table S6.** Average endocranial volume (ECV) in ml and as a proportion of body mass.

**Table S1.** Comprehensive list of studies identified across all search strategies for the systematic review of inhibitory control in non‐human animals.

**Table S2.** List of the literature used in this review, with the domain of inhibitory control tested, the task, the animal classes, the authors and the name of the species.
