# Supplementary material for: Inhibitory control tests in non‐human animals: validity, reliability, and perspectives
Source: Biol Rev Camb Philos Soc. 2025 Jul 28;100(6):2482–507. doi: 10.1111/brv.70055 (PMC12586309; doi:10.1111/brv.70055)
Supplement: Supplementary file 1 — Data S1. Supplementary Information. Table S3. Accuracy (% correct responses out of the total number of trials) for different tasks of inhibitory control, across different animal taxa. Table S4. Subject‐ and task‐related factors that can influence inhibitory control performance. Table S5. Mean, median and standard deviation (S.D.) number of subjects in tests (N) for which the sample size was recorded. Table S6. Average endocranial volume (ECV) in ml and as a proportion of body mass. [file BRV-100-2482-s002.docx]

**Supplementary materials**

**Table S1.** (see separate file, TableS1.xlsx). Comprehensive list of studies identified across all search strategies for the systematic review of inhibitory control in non-human animals. This table details the studies retrieved through systematic database searches (*ISI Web of Science*, *Pubmed* and *Google Scholar*). Each paper was screened (see methods section) and the table indicates whether it was included or excluded, and, if excluded, the reason why. All studies listed met the inclusion criteria established for the review.

**Table S2.** (see separate file, TableS2.xlsx). List of the literature used in this review with the domain of inhibitory control tested, the task, the animal classes, the authors and the name of the species. We selected articles containing at least one task of inhibitory control (from one domain of inhibitory control: inhibition of action, of a cognitive set, of a distraction or social inhibition), in at least one non-human animal species.

**Table S3.** Accuracy (% correct responses out of the total number of trials for different tasks of inhibitory control, across different animal taxa. Mean accuracy, standard deviation (SD), number of papers for which the accuracy of the task was available (*N*) and the standard error of measurement (SEM) are provided for each task. When an environmental factor was tested, only data from the control condition was included.

|  | **Detour task** | | | | **A-not-B task** | | | | **Cylinder task** | | | | **Middle cup task** | | | | **Plexiglas hole task** | | | | **Swing door task** | | | |
| --- | --- | --- | --- | --- | --- | --- | --- | --- | --- | --- | --- | --- | --- | --- | --- | --- | --- | --- | --- | --- | --- | --- | --- | --- |
|  | **Mean** | **SD** | ***N*** | **SEM** | **Mean** | **SD** | ***N*** | **SEM** | **Mean** | **SD** | ***N*** | **SEM** | **Mean** | **SD** | ***SEM*** | **N** | **Mean** | **SD** | ***N*** | **SEM** | **Mean** | **SD** | ***N*** | **SEM** |
| All animal classes | 57.13 | 24.94 | 19 | 5.72 | 57.29 | 32.58 | 44 | 4.91 | 58.27 | 25.37 | 74 | 2.95 | 44.7 | 26 | 8.12 | 11 | 65.7 | 37 | 6 | 15 | 18 | 22 | 14 | 5.8 |
| Great apes |  |  |  |  | 95.21 | 6.98 | 8 | 2.47 | 97.83 | 3 | 3 | 174 | 59 | 27.49 | 13.74 | 6 | 93.8 | 6.3 | 3 | 3.6 | 24.19 | 22.8 | 10 | 6.7 |
| Primates (excluding great apes) |  |  |  |  | 54.07 | 28.57 | 24 | 5.96 | 47.51 | 27.82 | 23 | 5.80 | 36.5 | 23.82 |  | 9 | 37.5 | 31 | 3 | 18 | 2.5 | 5 | 4 | 2.5 |
| Dogs | 75.71 | 14.43 | 9 | 6.45 | 72.35 | 22.72 | 8 | 11.36 | 72.87 | 12.02 | 11 | 3.80 |  |  |  |  |  |  |  |  |  |  |  |  |
| Carnivores (excluding dogs) | 71.66 | 12.25 | 4 | 8.66 | 28.60 |  | 1 |  | 79.79 | 10.52 | 7 | 4.29 |  |  |  |  |  |  |  |  |  |  |  |  |
| Mammals (excluding carnivores and primates) | 60.58 | 33.01 | 7 | 19.06 | 24.94 | 29.39 | 6 | 14.69 | 66.07 | 3.34 | 3 | 1.86 |  |  |  |  |  |  |  |  |  |  |  |  |
| Corvids and parrots |  |  |  |  | 14.47 | 3.87 | 10 | 2.23 | 65.62 | 23.66 | 12 | 7.13 |  |  |  |  |  |  |  |  |  |  |  |  |
| Birds (excluding corvids and parrots) | 21.35 | 27.79 | 7 | 19.65 | 54.50 |  | 1 |  | 49.47 | 23.11 | 15 | 7.70 |  |  |  |  |  |  |  |  |  |  |  |  |
| Fishes | 48.46 | 18.20 | 13 | 7.46 |  |  |  |  | 34.60 | 7.73 | 5 | 3.45 |  |  |  |  |  |  |  |  |  |  |  |  |
| Reptiles |  |  |  |  |  |  |  |  | 34.45 | 6.93 | 5 | 3.10 |  |  |  |  |  |  |  |  |  |  |  |  |
| Invertebrates | 80 |  | 1 |  |  |  |  |  |  |  |  |  |  |  |  |  |  |  |  |  |  |  |  |  |

**Table S4.** Subject- and task-related factors that can influence inhibitory control performance.

| **Influencing factors** | | | **Effect on inhibitory control performance** | **Task** | **Animal class** | **References** |
| --- | --- | --- | --- | --- | --- | --- |
| 1. **Context of the task** | | | | | | |
| **Task modalities** | | Larger opening in apparatus | Positive | modified cylinder | Felines | Bobrowicz & Osvath (2018) |
|  |  | Outward detour compared to inward detour | Positive | detour | Canines | Pongrácz *et al*. 2001 |
|  |  | Traffic noise *vs* no noise | Negative | cylinder | Birds | Templeton *et al*. 2023 |
|  |  | Larger size of the apparatus | Positive | cylinder | Felines | Bobrowicz & Osvath (2018) |
| **Salience of reward** | | Larger distance to the reward/barrier | Negative | detour | Rodents | Juszczak & Miller (2016) |
|  |  | Food odour vs no odour | No effect | cylinder | Fish | Santacà *et al*. (2019*b*) |
|  |  | Increased reward saliency | Negative | detour | Canines | Brucks *et al*. (2017) |
| **Presence of a conspecific** | | Alone vs with a partner | Positive | modified Stroop | Primates | Huguet *et al*. (2014) |
| 1. **Individuals factors** | | | | | | |
| **Experience** | Prior experience with transparent objects | | Positive | detour, cylinder | Birds | van Horik *et al*. (2018*b*, 2019) |
|  |  |  | No effect | cylinder | Fish | Santacà *et al*. (2019*b*) |
|  | Hand-tracking training | | Positive | A-not-B | Birds | Jelbert *et al*. (2016) |
|  | Previous experience with similar task | | Positive | detour, cylinder | Birds | van Horik *et al*. (2018*b*) |
| ***Morphology*** | Better body condition | | Positive | detour | Birds | Shaw (2017) |
|  |  |  | Negative | cylinder | Reptiles | Szabo *et al*. (2020) |
|  | Larger absolute brain size | | Positive | cylinder | Primates, canines | MacLean *et al*. (2014); Horschler *et al*. (2019) |
| ***Personality*** | More fearful temperament | | Negative | attention distraction | Primates | Bethell *et al*. (2019) |
|  | Bolder | | Positive | cylinder | Fishes | Lucon-Xiccato *et al*. (2020*a*) |
|  | Higher level of anxiety | | Negative | attention distraction | Primates | Allritz *et al*. (2016); Bethell *et al*. (2012) |
|  |  |  | Positive | reversal learning, cylinder | Primates | Damerieus *et al*. (2017) |
|  | Higher curiosity level | | Negative | reversal learning | Canines | Tapp *et al*. (2003) |
|  | More proactive (*vs* reactive) | | Positive | detour | Birds | Gomes *et al*. (2020) |
| ***Age and sex*** | Older individuals | | Negative | reversal learning | Primates | Bonté *et al*. (2014) |
|  |  |  | Negative | reversal learning | Primates | Kumpan *et al*. (2020) |
|  |  |  | Negative | reversal learning | Primates | Tsuchida *et al*. (2002) |
|  |  |  | Negative | reversal learning | Rodents | Schoenbaum *et al*. (2002) |
|  |  |  | Negative | reversal learning | Canines | Bray *et al*. (2014) |
|  |  |  | Negative | reversal learning | Canines | Wallis *et al*. (2016) |
|  |  |  | Negative | reversal learning | Canines | Tapp *et al*. (2003) |
|  | Males (compared to females) | | No effect | attention distraction | Primates | Boggiani *et al*. (2018) |
|  |  |  | Negative | attention distraction | Primates | Loyant *et al*. (2021) |
|  |  |  | Positive | detour | Fishes | Lucon-Xiccato *et al*. (2020*b*) |
| 1. **Environmental factors** | | | | | | |
| ***Social environment*** | Larger group size | | Positive | cylinder, reversal learning | Birds | Ashton *et al*. (2018) |
|  |  |  | Positive | cylinder | Canines | Johnson-Ulrich & Holekamp (2020) |
|  | Higher social tolerance | | Positive | middle cup | Primates | Joly *et al*. (2017) |
| ***Developmental environment*** | More unpredictable environment | | Positive | detour | Birds | van Horik *et al*. (2019) |
|  | Shelter dog *vs* domestic dog | | Negative | A-not-B | Canines | Fagnani *et al*. (2016) |
|  | Domesticated *vs* wild | | Positive | cylinder | Canines | Marshall-Pescini *et al*. (2015) |
|  |  |  | Negative | detour | Canines | Marshall-Pescini *et al*. (2016) |
|  | Wild *vs* captive | | Positive | detour | Fishes | Gatto *et al*. (2018) |
| ***Ecological factors*** | Lower food competition | | Positive | modified cylinder | Primates | Vlamings *et al*. (2010) |
|  | Higher fission–fusion | | Positive | A-not B, detour reaching, middle cup | Primates | Amici *et al*. (2008, 2018) |
|  | More diverse diet | | Positive | A-not-B, cylinder | 36 different species | Maclean *et al*. (2014) |

**Table S5.** Mean, median, and standard deviation (SD) number of subjects in tests (*N*) for which the sample size was recorded. Where possible, we used the sample size from analyses of subjects that completed the study.

|  | **Mean** | **Median** | **SD** | ***N*** |
| --- | --- | --- | --- | --- |
| **All animal classes and species** | 60.22 | 12 | 404.91 | 339 |
| **great apes** | 12.6 | 7 | 18.9 | 44 |
| **primates (excluding great apes)** | 11.4 | 10 | 6.08 | 109 |
| **dogs** | 357 | 30 | 1135 | 41 |
| **carnivores**  **(excluding dogs)** | 14.3 | 11 | 13.5 | 19 |
| **mammals (excluding carnivores and primates)** | 25.6 | 21 | 18.7 | 25 |
| **corvids and parrots** | 9.78 | 7 | 9,81 | 32 |
| **birds (excluding corvids and parrots)** | 49.3 | 31 | 60.0 | 38 |
| **reptiles** | 14.4 | 13 | 5.56 | 7 |
| **fish** | 33.7 | 27 | 24.3 | 24 |
| **invertebrates** | 49 | 54 | 33.8 | 3 |

**Table S6.** Average endocranial volume (ECV) in ml and as a proportion of body mass. Data from Maclean *et al*. (2014) and Kabadayi *et al*. (2016). *N* = number of species.

|  | **Great apes**  **(*N* = 4)** | **Carnivores (*N* = 3)** | **Primates (excluding great apes)**  **(*N* = 20)** | **Birds (*N* = 7)** | **Asian elephants (*N* = 1)** |  |
| --- | --- | --- | --- | --- | --- | --- |
| ECV (ml) | 394.358 | 94.800 | 57.010 | 6.384 | 4752.25 |  |
| ECV in ml/body mass in g | 0.007 | 0.004 | 0.015 | 0.0201 | 0.002 |  |

**References**

Abramson, J. Z., Paulina Soto, D., Beatriz Zapata, S., & Lloreda, M. V. H. (2018). Spatial perseveration error by alpacas (*Vicugna pacos*) in an A-not-B detour task. *Animal Cognition* **21**, 433–439.

Allritz, M., Call, J. & Borkenau, P. (2016). How chimpanzees (*Pan troglodytes*) perform in a modified emotional Stroop task. *Animal Cognition* **19**, 435–449.

Amici, F., Aureli, F. & Call, J. (2008). Fission-fusion dynamics, behavioral flexibility, and inhibitory control in primates. *Current Biology* **18**, 1415–1419.

Amici, F., Call, J., Watzek, J., Brosnan, S. & Aureli, F. (2018). Social inhibition and behavioural flexibility when the context changes: A comparison across six primate species. *Scientific Reports* **8**, 1–9.

Ashton, B. J., Ridley, A. R., Edwards, E. K. & Thornton, A. (2018). Cognitive performance is linked to group size and affects fitness in Australian magpies. *Nature* **554**, 364–367.

Banerjee, K., Chabris, C. F., Johnson, V. E., Lee, J. J., Tsao, F., & Hauser, M. D. (2009). General intelligence in another primate: individual differences across cognitive task performance in a New World monkey (*Saguinus oedipus*). *PLoS One* **4**, e5883.

Barrera, G., Alterisio, A., Scandurra, A., Bentosela, M., & D’Aniello, B. (2019). Training improves inhibitory control in water rescue dogs. *Animal Cognition* **22**, 127–131.

Beran, M. J., Washburn, D. A. & Rumbaugh, D. M. (2007). A Stroop-like effect in color-naming of color-word lexigrams by a chimpanzee (Pan troglodytes). *The Journal of General Psychology* **134**, 217–228.

Bethell, E. J., Cassidy, L. C., Brockhausen, R. R. & Pfefferle, D. (2019). Toward a standardized test of fearful temperament in primates: a sensitive alternative to the human intruder task for laboratory-housed rhesus macaques (Macaca mulatta). *Frontiers in psychology* **10**, 1051.

Bethell, E. J., Holmes, A., MacLarnon, A. & Semple, S. (2012). Evidence That Emotion Mediates Social Attention in Rhesus Macaques. *PLoS One* **7**, e44387.

Bobrowicz, K., & Osvath, M. (2018). Cats parallel great apes and corvids in motor self-regulation-not brain but material size matters. *Frontiers in Psychology* **9**, 1995.

Boggiani, L., Addessi, E. & Schino, G. (2018). Receiving aggression triggers attention bias in tufted capuchin monkeys. *Animal behaviour* **146**, 173–180.

Bond, A. B., Kamil, A. C. & Balda, R. P. (2007). Serial reversal learning and the evolution of behavioral flexibility in three species of North American corvids (Gymnorhinus cyanocephalus, Nucifraga columbiana, Aphelocoma californica). *Journal of Comparative Psychology* **121**, 372–379.

Bonté, E., Kemp, C. & Fagot, J. (2014). Age effects on transfer index performance and executive control in baboons (*Papio papio*). *Frontiers in psychology* **5**, 188.

Boogert, N. J., Anderson, R. C., Peters, S., Searcy, W. A. & Nowicki, S. (2011). Song repertoire size in male song sparrows correlates with detour reaching, but not with other cognitive measures. *Animal Behaviour* **81**, 1209–1216.

Brandão, M. L., de Almeida Fernandes, A. M. T. & Gonçalves-de-Freitas, E. (2019). Male and female cichlid fish show cognitive inhibitory control ability. *Scientific Reports* *9*, 157951.

Bray, E. E., Gruen, M. E., Gnanadesikan, G. E., Horschler, D. J., Levy, K. M., Kennedy, B. S., Hare, B.A. & MacLean, E. L. (2021). Dog cognitive development: a longitudinal study across the first 2 years of life. *Animal Cognition* **24**, 311–328.

Bray, E. E., MacLean, E. L. & Hare, B. A. (2014). Context specificity of inhibitory control in dogs. *Animal Cognition* **17**, 15–31.

Bridgeman, J. M. & Tattersall, G. J. (2019). Tortoises develop and overcome position biases in a reversal learning task. *Animal Cognition* **22**, 265–275.

Brucks, D., Marshall-Pescini, S. & Range, F. (2019). Dogs and wolves do not differ in their inhibitory control abilities in a non-social test battery. *Animal Cognition* **22**, 1–15.

Brucks, D., Marshall-Pescini, S., Wallis, L. J., Huber, L. & Range, F. (2017). Measures of dogs’ inhibitory control abilities do not correlate across tasks*. Frontiers in Psychology* **8**, 1–17.

Campos, H. C., Debert, P., Barros, R. D. S. & McIlvane, W. J. (2011). Relational discrimination by pigeons in a go/no‐go procedure with compound stimuli: a methodological note. *Journal of the Experimental Analysis of Behavior* **96**, 417–426.

Cantwell, A., Buckholtz, J. W., Atencia, R. & Rosati, A. G. (2022). The origins of cognitive flexibility in chimpanzees. *Developmental Science* **25**, e13266.

Cauchoix, M., Hermer, E., Chaine, A. S. & Morand-Ferron, J. (2017). Cognition in the field: Comparison of reversal learning performance in captive and wild passerines*. Scientific Reports* **7**, 12945.

Cavalli, C. M., Carballo, F., Dzik, M. V., Underwood, S., & Bentosela, M. (2018). Are animal-assisted activity dogs different from pet dogs? A comparison of their sociocognitive abilities. *Journal of Veterinary Behavior* **23**, 76–81.

Chandra, S. B., Hosler, J. S. & Smith, B. H. (2000). Heritable variation for latent inhibition and its correlation with reversal learning in honeybees (*Apis mellifera*). *Journal of Comparative Psychology* **114**, 86.

Chow, P. K. Y., Leaver, L. A., Wang, M. & Lea, S. E. (2017). Touch screen assays of behavioural flexibility and error characteristics in Eastern grey squirrels (*Sciurus carolinensis). Animal Cognition* **20**, 459–471.

Cole, E., Chad, M., Moman, V. & Mumby, D. G. (2020). A Go/No-go delayed nonmatching-to-sample procedure to measure object-recognition memory in rats. *Behavioural Processes*, **178**, 104180.

Cook, P. F., Spivak, M. & Berns, G. (2016). Neurobehavioral evidence for individual differences in canine cognitive control: An awake fMRI study. *Animal cognition* **19**, 867–878.

Coomes, J. R., Davidson, G. L., Reichert, M. S., Kulahci, I. G., Troisi, C. A. & Quinn, J. L. (2022). Inhibitory control, exploration behaviour and manipulated ecological context are associated with foraging flexibility in the great tit. *Journal of Animal Ecology* **91**, 320-333.

Cross, F. R. & Jackson, R. R. (2016). The execution of planned detours by spider‐eating predators. *Journal of the experimental analysis of behavior* **105**, 194–210.

Damerius, L. A., Graber, S. M., Willems, E. P. & van Schaik, C. P. (2017). Curiosity boosts orang-utan problem-solving ability. *Animal Behaviour* **134**, 57–70.

Davidson, G. L., Reichert, M. S., Coomes, J. R., Kulahci, I. G., de la Hera, I. & Quinn, J. L. (2022). Inhibitory control performance is repeatable over time and across contexts in a wild bird population. *Animal Behaviour* **187**, 305–318.

Dolzani, S. D., Nakamura, S. & Cooper, D. C. (2013). A novel variable delay Go/No-Go task to study attention, motivation and working memory in the head-fixed rodent. *F1000Research* **2**, 125

Fagnani, J., Barrera, G., Carballo, F. & Bentosela, M. (2016). Is previous experience important for inhibitory control? A comparison between shelter and pet dogs in A-not-B and cylinder tasks. *Animal Cognition* **19**, 1165–1172.

Ferreira, V. H. B., Reiter, L., Germain, K., Calandreau, L., & Guesdon, V. (2020). Uninhibited chickens: ranging behaviour impacts motor self-regulation in free-range broiler chickens (*Gallus gallus domesticus*). *Biology Letters* **16**, 20190721.

Fiske, J. C. & Potter, G. D. (1979). Discrimination reversal learning in yearling horses. *Journal of Animal Science* **49**, 583–588.

Forss, S. I., Willems, E., Call, J. & van Schaik, C. P. (2016). Cognitive differences between orang-utan species: a test of the cultural intelligence hypothesis. *Scientific reports* ***6***, 30516.

Freeman, L. R., & Aston-Jones, G. (2020). Activation of medial hypothalamic orexin neurons during a Go/No-Go task. *Brain research* **1731**, 145928.

Garnham, L. C., Clarke, C.,& Løvlie, H. (2022). How inhibitory control relates to positive and negative affective states in red junglefowl. *Frontiers in Veterinary Science*, **9**, 872487.

Gatto, E., Lucon-Xiccato, T., & Bisazza, A. (2018). Factors affecting the measure of inhibitory control in a fish (*Poecilia reticulata*). *Behavioural processes* **57**, 11–17.

Ghasemian, S., Vardanjani, M. M., Sheibani, V. & Mansouri, F. A. (2021). Dimensional bias and adaptive adjustments in inhibitory control of monkeys. *Animal Cognition* **24**, 815–828.

Gnanadesikan, G. E., Hare, B., Snyder-Mackler, N., & MacLean, E. L. (2020). Estimating the heritability of cognitive traits across dog breeds reveals highly heritable inhibitory control and communication factors. *Animal Cognition* **23**, 953–964.

Gomes, A. C. R., Guerra, S., Silva, P. A., Marques, C. I., Trigo, S., Boogert, N. J., & Cardoso, G. C. (2020). Proactive common waxbills make fewer mistakes in a cognitive assay, the detour-reaching task. *Behavioral Ecology and Sociobiology* **74**, 1–15.

Hauser, M. D., Santos, L. R., Spaepen, G. M. & Pearson, H. E. (2002). Problem solving, inhibition and domain-specific experience: experiments on cottontop tamarins, *Saguinus oedipus.* *Animal Behaviour* **64**, 387–396.

Herrmann, E., Call, J., Hernández-Lloreda, M. V., Hare, B. & Tomasello, M. (2007). Humans have evolved specialized skills of social cognition: The cultural intelligence hypothesis. S*cience* **317**, 1360–1366.

Hopper, L. M., Allritz, M., Egelkamp, C. L., Huskisson, S. M., Jacobson, S. L., Leinwand, J. G., & Ross, S. R. (2021). A comparative perspective on three primate species’ responses to a pictorial emotional stroop task. *Animals* **11**, 588

Horschler, D. J., Hare, B., Call, J., Kaminski, J., Miklósi, Á. & MacLean, E. L. (2019). Absolute brain size predicts dog breed differences in executive function. *Animal Cognition* **22**, 187–198.

Huguet, P., Barbet, I., Belletier, C., Monteil, J. M. & Fagot, J. (2014). Cognitive control under social influence in baboons. *Journal of Experimental Psychology: General* **143**, 2067–2073.

Isaksson, E., Urhan, A. U. & Brodin, A. (2018). High level of self-control ability in a small passerine bird. *Behavioral Ecology and Sociobiology* **72**, 118.

Jelbert, S. A., Taylor, A. H. & Gray, R. D. (2016). Does absolute brain size really predict self-control? Hand-tracking training improves performance on the A-not-B task. *Biology Letters* **12**, 20150871.

Johnson-Ulrich, L., Johnson-Ulrich, Z., & Holekamp, K. (2018). Proactive behavior, but not inhibitory control, predicts repeated innovation by spotted hyenas tested with a multi-access box. *Animal Cognition* **21**, 379–392.

Johnson-Ulrich, L. & Holekamp, K. E. (2020). Group size and social rank predict inhibitory control in spotted hyaenas. *Animal Behaviour* **160**, 157–168.

Johnsson, R. D., Veillet, P. S., Connelly, F., Endler, J. A., Roth, T. C. & Lesku, J. A. (2023). Wild Australian magpies learn to pull intact, not broken, strings to obtain food. *Behavioral Ecology and Sociobiology* **77**, 49.

Joly, M., Micheletta, J., De Marco, A., Langermans, J. A., Sterck, E. H. & Waller, B. M. (2017). Comparing physical and social cognitive skills in macaque species with different degrees of social tolerance. *Proceedings of the Royal Society B: Biological Sciences* **284**, 20162738.

Judge, P. G., Evans, D. W., Schroepfer, K. K., & Gross, A. C. (2011). Perseveration on a reversal-learning task correlates with rates of self-directed behavior in nonhuman primates. *Behavioural Brain Research* **222**, 57–65.

Junttila, S., Huohvanainen, S., & Tiira, K. (2021). Effect of sex and reproductive status on inhibitory control and social cognition in the domestic dog (*Canis familiaris*). *Animals* **11**, 2448.

Junttila, S., Valros, A., Mäki, K., Väätäjä, H., Reunanen, E., & Tiira, K. (2022). Breed differences in social cognition, inhibitory control& spatial problem-solving ability in the domestic dog (*Canis familiaris*). *Scientific Reports* **12**, 22529.

Juszczak, G. R. & Miller, M. (2016). Detour behavior of mice trained with transparent, semi-transparent and opaque barriers. *PloS* One **11**, e0162018.

Kabadayi, C., Jacobs, I. & Osvath, M. (2017*a*). The development of motor self-regulation in ravens. *Frontiers in Psychology* **8**, 2100.

Kabadayi, C., Krasheninnikova, A., O’Neill, L., van de Weijer, J., Osvath, M. & von Bayern, A. M. (2017*b*). Are parrots poor at motor self-regulation or is the cylinder task poor at measuring it? *Animal Cognition* **20**, 1137–1146.

Kabadayi, C., Taylor, L. A., von Bayern, A. M. & Osvath, M. (2016). Ravens, New Caledonian crows and jackdaws parallel great apes in motor self-regulation despite smaller brains. *Royal Society Open Science*, **3**, 160104.

King, H. M., Kurdziel, L. B., Meyer, J. S. & Lacreuse, A. (2012). Effects of testosterone on attention and memory for emotional stimuli in male rhesus monkeys. *Psychoneuroendocrinology* **37**, 396–409.

Knolle, F., Goncalves, R. P., Davies, E. L., Duff, A. R., & Morton, A. J. (2019). Response-inhibition during problem solving in sheep. *International Journal of Comparative Psychology* **32**.

Komischke, B., Giurfa, M., Lachnit, H. & Malun, D. (2002). Successive olfactory reversal learning in honeybees. *Learning & Memory* **9**, 122–129.

Krichbaum, S. & Lazarowski, L. (2022). Reward type affects dogs’ performance in the cylinder task. Animal *Behavior and Cognition* **9,** 287–297.

Kumpan, L. T., Smeltzer, E. A. & Teichroeb, J. A. (2020). Animal cognition in the field: performance of wild vervet monkeys (*Chlorocebus pygerythrus*) on a reversal learning task. *Animal Cognition* **23**, 523–534*.*

Lacreuse, A., Schatz, K., Strazzullo, S., King, H. M. & Ready, R. (2013). Attentional biases and memory for emotional stimuli in men and male rhesus monkeys. *Animal Cognition* **16**, 861–871.

Laméris, D. W., Verspeek, J., Eens, M. & Stevens, J. M. (2022). Social and nonsocial stimuli alter the performance of bonobos during a pictorial emotional Stroop task. *American Journal of Primatology* **84**, e23356.

Landman, R., Sharma, J., Sur, M. & Desimone, R. (2014). Effect of distracting faces on visual selective attention in the monkey. *Proceedings of the National Academy of Sciences* **29**, 1–6.

Langbein, J. (2018). Motor self-regulation in goats (*Capra aegagrus hircus*) in a detour-reaching task. *PeerJ* **6**, e5139.

Langley, E. J., Adams, G., Beardsworth, C. E., Dawson, D. A., Laker, P. R., van Horik, J. O., Whiteside, m. a., Wilson, a. j., & Madden, J. R. (2020). Heritability and correlations among learning and inhibitory control traits. *Behavioral Ecology* **31**, 798–806.

Laschober, M., Mundry, R., Huber, L., & Schwing, R. (2021). Kea (*Nestor notabilis*) show flexibility and individuality in within-session reversal learning tasks. *Animal Cognition* **24**, 1339–1351.

Liu, S., Heitz, R. P. & Bradberry, C. W. (2009). A touch screen based stop signal response task in rhesus monkeys for studying impulsivity associated with chronic cocaine self-administration. *Journal of neuroscience methods* **177**, 67–72.

Logan, C. J., McCune, K. B., MacPherson, M., Johnson-Ulrich, Z., Rowney, C., Seitz, B., Blaisdell, A.P., Deffner, D. & Wascher, C. A. F. (2022). Are the more flexible individuals also better at inhibition. *Animal Behaviour Cognition* **9**, 14–36.

López, J. C., Broglio, C., Rodrı́guez, F., Thinus-Blanc, C. & Salas, C. (2000). Reversal learning deficit in a spatial task but not in a cued one after telencephalic ablation in goldfish. *Behavioural Brain Research* **109**, 91–98.

Loyant, L., Waller, B. M., Micheletta, J. & Joly, M. (2021). Heterogeneity of performances in several inhibitory control tasks: male rhesus macaques are more easily distracted than females. *Royal Society Open Science* **8**, 211564.

Loyant, L., Waller, B.M., Micheletta, J., Meunier, H., Ballesta, S. & Joly, M. (2022). Validation of a battery of inhibitory control tasks reveals a multifaceted structure in non-human primates. *Peer J* **10**, e12863.

Loyant, L., Waller, B.M., Micheletta, J., Meunier, H., Ballesta, S. & Joly, M. (2023). Tolerant macaque species are less impulsive and reactive. *Animal Cognition* **26***,* 1453–146.

Lucon-Xiccato, T. & Bertolucci, C. (2019). Guppies show rapid and lasting inhibition of foraging behaviour. *Behavioural Processes* **164**, 91–99.

Lucon-Xiccato, T. & Bisazza, A. (2014). Discrimination reversal learning reveals greater female behavioural flexibility in guppies. *Biology Letters* **10**, 20140206.

Lucon-Xiccato, T., Bisazza, A. & Bertolucci, C. (2020*a*). Guppies show sex and individual differences in the ability to inhibit behaviour. *Animal Cognition* **23**, 535–543.

Lucon-Xiccato, T., Gatto, E., & Bisazza, A. (2017). Fish perform like mammals and birds in inhibitory motor control tasks. *Scientific Reports* **7**, 13144.

Lucon-Xiccato, T., Montalbano, G. & Bertolucci, C. (2020*b*). Personality traits covary with individual differences in inhibitory abilities in 2 species of fish. *Current Zoology* **66**, 187–195.

Lyons, D. M., Lopez, J. M., Yang, C., & Schatzberg, A. F. (2000). Stress-level cortisol treatment impairs inhibitory control of behavior in monkeys. *Journal of Neuroscience* **20**, 7816–7821.

Macario, A., Darden, S. K., Verbruggen, F., & Croft, D. P. (2021). Intraspecific variation in inhibitory motor control in guppies, *Poecilia reticulata*. *Journal of Fish Biology* **98**, 317–328.

MacKinlay, R. D., & Shaw, R. C. (2019). Male New Zealand robin (*Petroica longipes*) song repertoire size does not correlate with cognitive performance in the wild. *Intelligence* **74**, 25–33.

MacLean, E. L., Hare, B., Nun, C. L., Addess, E., Amic, F., Anderson, R. C., Aureli, F., Baker, J. M., Bania, A. E., Barnard, A. M., Boogert, N. J., Brannon, E. M., Bray, E. E., Bray, J., Brent, L. J. N., *et al*. (2014). The evolution of self-control. *Proceedings of the National Academy of Sciences of the United States of America* **111,** E2140–E2148.

MacLean, E. L., Herrmann, E., Suchindran, S. & Hare, B. (2017). Individual differences in cooperative communicative skills are more similar between dogs and humans than chimpanzees. *Animal Behaviour* **126**, 41–51.

MacLean, E. L., Sandel, A. A., Bray, J., Oldenkamp, R. E., Reddy, R. B. & Hare, B. A. (2013). Group size predicts social but not nonsocial cognition in lemurs. *PLoS One* **8**, e66359.

Marshall-Pescini, S., Frazzi, C., & Valsecchi, P. (2016). The effect of training and breed group on problem-solving behaviours in dogs. *Animal Cognition* **19**, 571–579.

Marshall-Pescini, S., Virányi, Z. & Range, F. (2015). The effect of domestication on inhibitory control: wolves and dogs compared. *PloS One* **10**, e0118469.

Massera, A., Bonaiuto, J. J., Gautier-Martins, M., Costa, S., Rayson, H. & Ferrari, P. F. (2023). Longitudinal effects of early psychosocial deprivation on macaque executive function: Evidence from computational modelling. *Proceedings of the Royal Society B* **290**, 20221993.

McCallum, E., & Shaw, R. C. (2023). Repeatability and heritability of inhibitory control performance in wild toutouwai (*Petroica longipes*). *Royal Society Open Science* **10,** 231476.

Meier, C., Pant, S. R., van Horik, J. O., Laker, P. R., Langley, E. J., Whiteside, M. A., Verbruggen, F. & Madden, J. R. (2017). A novel continuous inhibitory-control task: variation in individual performance by young pheasants (*Phasianus colchicus*). *Animal Cognition* **20,** 1035–1047.

Middlebrooks, P. G. & Schall, J. D. (2014). Response inhibition during perceptual decision making in humans and macaques. *Attention, Perception, & Psychophysics* **76**, 353–366.

Montalbano, G., Bertolucci, C., & Lucon-Xiccato, T. (2020). Measures of inhibitory control correlate between different tasks but do not predict problem-solving success in a fish, *Poecilia reticulata*. *Intelligence* **82**, 101486.

Morita, M., Nakahara, K. & Hayashi, T. (2004). A rapid presentation event-related functional magnetic resonance imaging study of response inhibition in macaque monkeys. *Neuroscience Letters* **356**, 203–206.

Müller, C. A., Riemer, S., Virányi, Z., Huber, L. & Range, F. (2016). Inhibitory control, but not prolonged object-related experience appears to affect physical problem-solving performance of pet dogs. *PloS One* **11**, e0147753.

Neiworth, J. J., Balaban, M. T., Wagner, K., Carlsen, A., Min, S., Kwon, Y. I. C. & Rieth, I. (2022). A modified version of the dimensional change card sort task tests cognitive flexibility in children (*Homo sapiens*) and cotton-top tamarins (*Saguinus oedipus*). *Journal of Comparative Psychology* **136**, 155-171

O'Hara, M., Huber, L., & Gajdon, G. K. (2015). The advantage of objects over images in discrimination and reversal learning by kea, Nestor notabilis. *Animal Behaviour* **101**, 51–60.Olsen, M. R. (2022). An investigation of two ostensibly inhibitory control tasks used in canine cognition. *Applied Animal Behaviour Science* **256**, 105770.

Osthaus, B., Proops, L., Hocking, I. & Burden, F. (2013) Spatial cognition and perseveration by horses, donkeys and mules in a simple A-not-B detour task. *Animal Cognition* **16**, 301–305

Paine, T. A., & Olmstead, M. C. (2004). Cocaine disrupts both behavioural inhibition and conditional discrimination in rats. *Psychopharmacology* **175**, 443–450.

Parker, K. J., Buckmaster, C. L., Justus, K. R., Schatzberg, A. F., & Lyons, D. M. (2005). Mild early life stress enhances prefrontal-dependent response inhibition in monkeys. *Biological Psychiatry* **57**, 848–855.

Pongrácz, P., Miklósi, Á., Kubinyi, E., Gurobi, K., Topál, J. & Csányi, V. (2001). Social learning in dogs: the effect of a human demonstrator on the performance of dogs in a detour task. *Animal Behaviour* **62**, 1109–1117.

Porter, J. N., Olsen, A. S., Gurnsey, K., Dugan, B. P., Jedema, H. P. & Bradberry, C. W. (2011). Chronic cocaine self-administration in rhesus monkeys: impact on associative learning, cognitive control, and working memory. *Journal of Neuroscience* **31**, 4926–4934.

Raoult, C. M. C., Osthaus, B., Hildebrand, A. C. G., McElligott, A. G. & Nawroth, C. (2021). Goats show higher behavioural flexibility than sheep in a spatial detour task. *Royal Society Open Science* **8**, 201627.

Rasolofoniaina, B., Kappeler, P. M. & Fichtel, C. (2021). Wild narrow‐striped mongooses use social information to enhance behavioural flexibility. *Ethology* **127**, 253–266.

Reddy, R. B., MacLean, E. L., Sandel, A. A. & Hare, B. (2015). Social inhibitory control in five lemur species. *Primates* **56**, 241–252

Rochais, C., Schradin, C. & Pillay, N. (2023). Cognitive performance is linked to survival in free-living African striped mice. *Proceedings of the Royal Society* *B* **290**, 20230205.

Rudolph, K. & Fichtel, C. (2017). Inhibitory control in douc langurs (*Pygathrix nemaeus and P. cinerea*). Vietnamese Journal of Primatology **2**, 73–81.

Ryding, S., Garnham, L. C., Abbey-Lee, R. N., Petkova, I., Kreshchenko, A., & Løvlie, H. (2021). Impulsivity is affected by cognitive enrichment and links to brain gene expression in red junglefowl chicks. *Animal Behaviour* **178**, 195–207.

Santacà, M., Busatta, M., Lucon-Xiccato, T. & Bisazza, A. (2019*a*). Sensory differences mediate species variation in detour task performance. *Animal Behaviour* **155**, 153–162.

Santacà, M., Busatta, M., Savaşçı, B. B., Lucon-Xiccato, T., & Bisazza, A. (2019*b*). The effect of experience and olfactory cue in an inhibitory control task in guppies, Poecilia reticulata. *Animal Behaviour* **151**, 1–7.

Savaşçı, B. B., Lucon-Xiccato, T., & Bisazza, A. (2021). Ontogeny and personality affect inhibitory control in guppies, *Poecilia reticulata*. *Animal Behaviour* **180**, 111–121.

Schmitt, V., Pankau, B. & Fischer, J. (2012). Old world monkeys compare to apes in the primate cognition test battery. *PloS One* **7**, e32024.

Schoenbaum, G., Nugent, S., Saddoris, M. P. & Gallagher, M. (2002). Teaching old rats new tricks: Age-related impairments in olfactory reversal learning. *Neurobiology of Aging* **23**, 555–564.

Shajid Pyari, M., Vékony, K., Uccheddu, S., & Pongrácz, P. (2022). Companion cats show no effect of trial-and-error learning compared to dogs in a transparent-obstacle detour task. *Animals***13***, 32.*

Shaw, R. C. (2017). Testing cognition in the wild: factors affecting performance and individual consistency in two measures of avian cognition. *Behavioural Processes* **134**, 31–36.

Shibasaki, M. & Kawai, N. (2009). Rapid detection of snakes by Japanese monkeys (*Macaca fuscata*): an evolutionarily predisposed visual system. *Journal of Comparative Psychology* **123**, 131-135.

Smith, B. P. & Litchfield, C. A. (2010). How well do dingoes, *Canis dingo*, perform on the detour task?. *Animal Behaviour* **80**, 155–162.

Sollis, J. G., Ashton, B. J., Speechley, E. M., & Ridley, A. R. (2023). Repeated testing does not confound cognitive performance in the Western Australian magpie (*Cracticus tibicen dorsalis*). *Animal Cognition* **26**, 579–588.

Soravia, C., Ashton, B. J., Thornton, A. & Ridley, A. R. (2022). General cognitive performance declines with female age and is negatively related to fledging success in a wild bird. *Proceedings of the Royal Society B* 289, 20221748.

Sovrano, V. A., Baratti, G., & Potrich, D. (2018). A detour task in four species of fishes. *Frontiers in Psychology* **9**, 2341.

Stanton, L. A., Bridge, E. S., Huizinga, J., Johnson, S. R., Young, J. K. & Benson-Amram, S. (2021). Variation in reversal learning by three generalist mesocarnivores. *Animal Cognition* **24**, 555–568.

Stenfelt, J., Yngvesson, J., Blokhuis, H. J. & Rørvang, M. V. (2022). Dairy cows did not rely on social learning mechanisms when solving a spatial detour task. *Frontiers in Veterinary Science* **9**, 956559.

Stow, M. K., Vernouillet, A., & Kelly, D. M. (2018). Neophobia does not account for motoric self-regulation performance as measured during the detour-reaching cylinder task. *Animal Cognition* **21**, 565–574.

Szabo, B., Hoefer, S. & Whiting, M. J. (2020). Are lizards capable of inhibitory control? Performance on a semi-transparent version of the cylinder task in five species of Australian skinks. *Behavioral Ecology and Sociobiology* **74**, 1–15.

Szabo, B., Noble, D. W., & Whiting, M. J. (2019). Context-specific response inhibition and differential impact of a learning bias in a lizard. *Animal Cognition* **22**, 317–329.

Tapp, P. D., Siwak, C. T., Estrada, J., Head, E., Muggenburg, B. A., Cotman, C. W. & Milgram, N. W. (2003). Size and reversal learning in the beagle dog as a measure of executive function and inhibitory control in aging. *Learning and Memory* **10**, 64–73.

Templeton, C. N., O’Connor, A., Strack, S., Meraz, F., & Herranen, K. (2023). Traffic noise inhibits inhibitory control in wild-caught songbirds. *IScience* **26**, 106650.

Tremblay, L. & Schultz, W. (2000). Reward-related neuronal activity during go-nogo task performance in primate orbitofrontal cortex. *Journal of Neurophysiology* **83**, 1864–1876.

Triki, Z., & Bshary, R. (2021). Sex differences in the cognitive abilities of a sex-changing fish species *Labroides dimidiatus*. *Royal Society Open Science* **8**, 210239.

Tsuchida, J., Kubo, N. & Kojima, S. (2002). Position reversal learning in aged Japanese macaques*. Behavioural Brain Research* **129**, 107–112.

van Horik, J. O., Beardsworth, C. E., Laker, P. R., Langley, E. J. G., Whiteside, M. A. & Madden, J. R. (2019). Unpredictable environments enhance inhibitory control in pheasants. *Animal Cognition* **22**, 1105–1114.

van Horik, J. O., Beardsworth, C. E., Laker, P. R., Whiteside, M. A. & Madden, J. R. (2020). Response learning confounds assays of inhibitory control on detour tasks. *Animal Cognition* **23**, 215–225.

van Horik, J.O., Langley, E.J., Whiteside, M.A., Laker, P.R., Beardsworth, C.E. & Madden, J.R. (2018*a*). Do detour tasks provide accurate assays of inhibitory control? *Proceedings of the Royal Society B: Biological Sciences* **285**, 20180150.

van Horik, J. O., Langley, E. J., Whiteside, M. A., Laker, P. R. & Madden, J. R. (2018*b*). Intra-individual variation in performance on novel variants of similar tasks influences single factor explanations of general cognitive processes. *Royal Society Open Science* **5**, 171919.

Vernouillet, A., Anderson, J., Clary, D., & Kelly, D. M. (2016). Inhibition in Clark’s nutcrackers (*Nucifraga columbiana*): results of a detour-reaching test. *Animal Cognition* **19**, 661–665.

Vernouillet, A. A. A., Stiles, L. R., Andrew McCausland, J. & Kelly, D. M. (2018). Individual performance across motoric self-regulation tasks are not correlated for pet dogs. *Learning and Behavior* **46**, 522–536.

Vlamings, P. H. J. M., Hare, B. & Call, J. (2010). Reaching around barriers: The performance of the great apes and 3-5-year-old children. *Animal Cognition* **13**, 273–285.

Völter, C. J., Tinklenberg, B., Call, J. & Seed, A. M. (2022). Inhibitory control and cue relevance modulate chimpanzees’(*Pan troglodytes*) performance in a spatial foraging task. *Journal of Comparative Psychology* **136**, 105-120.

Vonk, J., McGuire, M. & Leete, J. (2022). Testing for the “Blues”: Using the Modified Emotional Stroop Task to Assess the Emotional Response of Gorillas. *Animals* **12**, 1188.

Wallis, L. J., Virányi, Z., Müller, C. A., Serisier, S., Huber, L . & Range, F. (2016). Aging effects on discrimination learning, logical reasoning and memory in pet dogs. *Age* **38**, 1–18.

Wang, L., Luo, Y., Lin, H., Xu, N., Gu, Y., Bu, H. & Li, Z. (2023). Performance on inhibitory tasks does not relate to handedness in several small groups of callitrichids. *Animal Cognition* **26**, 415–423.

Wascher, C. A., Allen, K. & Szipl, G. (2021). Learning and motor inhibitory control in crows and domestic chickens. *Royal Society Open Science* **8**, 210504.

Workman, K. P., Healey, B., Carlotto, A., & Lacreuse, A. (2019). One‐year change in cognitive flexibility and fine motor function in middle‐aged male and female marmosets (*Callithrix jacchus*). *American Journal of Primatology* **81**, e22924.
